# Teste Cardiopulmonar em Pacientes Pós-COVID-19: De Onde vem a Intolerância ao Exercício?

**DOI:** 10.36660/abc.20220150

**Published:** 2023-02-16

**Authors:** Mauricio Milani, Juliana Goulart Prata Oliveira Milani, Graziella França Bernardelli Cipriano, Lawrence Patrick Cahalin, Ricardo Stein, Gerson Cipriano

**Affiliations:** 1 Fitcordis Medicina do Exercício Brasília DF Brasil Fitcordis Medicina do Exercício , Brasília , DF – Brasil; 2 Universidade de Brasília Programa de Pós-Graduação em Ciências e Tecnologias da Saúde Brasília DF Brasil Universidade de Brasília - Programa de Pós-Graduação em Ciências e Tecnologias da Saúde , Brasília , DF – Brasil; 3 Universidade de Brasília Programa de Ciências da Reabilitação Brasília DF Brasil Universidade de Brasília - Programa de Ciências da Reabilitação , Brasília , DF – Brasil; 4 Department of Physical Therapy University of Miami Miller School of Medicine Florida EUA Department of Physical Therapy , University of Miami , Miller School of Medicine , Florida – EUA; 5 Universidade Federal do Rio Grande do Sul Programa de Pós-Graduação em Cardiologia e Ciências Cardiovasculares Porto Alegre RS Brasil Universidade Federal do Rio Grande do Sul – Programa de Pós-Graduação em Cardiologia e Ciências Cardiovasculares , Porto Alegre , RS – Brasil

**Keywords:** COVID-19, Teste de Esforço, Aptidão Cardiorrespiratória

## Abstract

**Fundamento:**

A intolerância ao exercício pós-COVID-19 não é bem entendida. O teste de esforço cardiopulmonar (TECP) pode identificar as limitações ao exercício subjacentes.

**Objetivos:**

Avaliar a etiologia e a magnitude da intolerância ao exercício em sujeitos pós-COVID-19.

**Métodos:**

Estudo de coorte que avaliou sujeitos com níveis de gravidades diferentes da doença COVID-19 e um grupo de controle selecionado por pareamento por escores de propensão. Em uma amostra seleta com TECP anterior à infecção viral disponível, foram realizadas comparações antes e depois. O nível de significância foi de 5% em toda a análise.

**Resultados:**

Foram avaliados cento e quarenta e dois sujeitos com COVID-19 (idade mediana: 43 anos, 57% do sexo masculino), com níveis de gravidade de doença diferentes (60% leve, 21% moderada, 19% grave). O TECP foi realizado 11,5 (7,0, 21,2) semanas após o aparecimento da doença, com as limitações ao exercício sendo atribuídas aos sistemas muscular periférico (92%), pulmonar (6%), e cardiovascular (2%). Menor valor mediano do consumo de oxigênio pico percentual foi observado no subgrupo com níveis graves de doença (72,2%) em comparação com os controles (91,6%). O consumo de oxigênio foi diferente entre os grupos com diferentes níveis de gravidade de doença e o controle no pico e nos limiares ventilatórios. Inversamente, os equivalentes ventilatórios, a inclinação da eficiência do consumo de oxigênio, e o pico do pulso de oxigênio foram semelhantes. A análise do subgrupo de 42 sujeitos com TECP prévio revelou uma redução significativa no pico de velocidade da esteira no subgrupo com nível leve de doença, e no consumo de oxigênio no pico e nos limiares ventilatórios nos subgrupos com níveis moderado/grave. Por outro lado, os equivalentes ventilatórios, a inclinação da eficiência do consumo de oxigênio e o pico do pulso de oxigênio não apresentaram alterações significativas.

**Conclusões:**

A fadiga do músculo periférico foi a etiologia de limitação de exercício mais comum em pacientes pós-COVID-19 independentemente da gravidade da doença. Os dados sugerem que o tratamento deve enfatizar programas de reabilitação abrangentes, incluindo componentes aeróbicos e de fortalecimento muscular.

**Figura Central:**
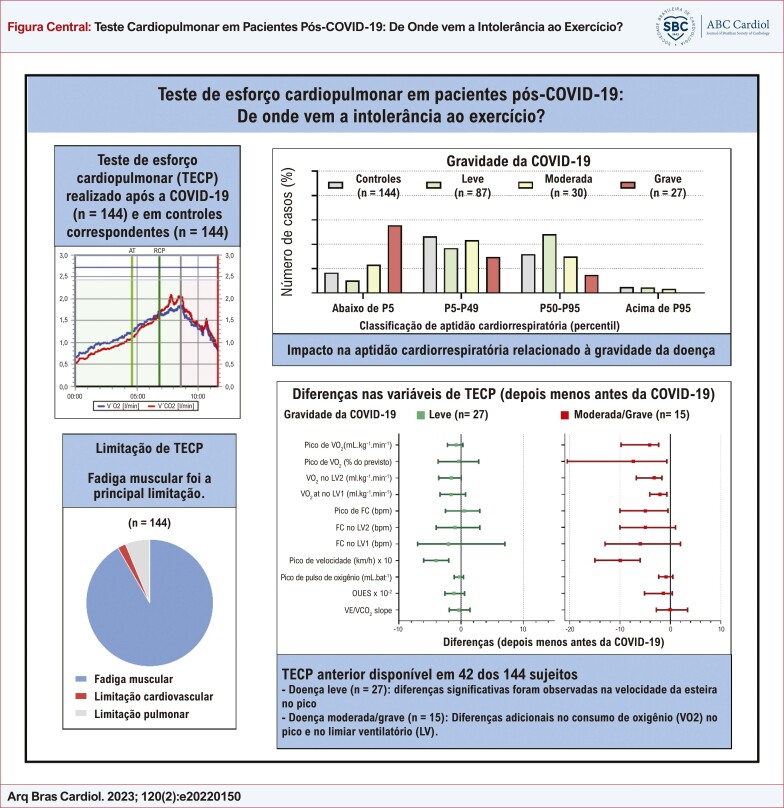
: Teste Cardiopulmonar em Pacientes Pós-COVID-19: De Onde vem a Intolerância ao Exercício?

## Introdução

A COVID-19 é uma doença multissistêmica com manifestações agudas que variam de assintomáticas a críticas. ^1^ A limitação ventilatória na espirometria foi descrita em um terço ou menos dos sujeitos na alta hospitalar, ^2 , 3^ com incidência mais baixa no acompanhamento de médio prazo. ^4 , 5^ Inversamente, anormalidades na difusão foram descritas em maiores proporções, especialmente em indivíduos com níveis mais graves da doença. ^3 , 4 , 6^ Além disso, muitos casos de injúria miocárdica também foram descritos em sujeitos afetados mais gravemente. ^7 , 8^ Ademais, após a recuperação da fase aguda, há cada vez mais relatos de sintomas persistentes, e essa condição clínica é chamada de “síndrome aguda pós-COVID-19” ou “COVID longa,”. ^1 , 9^ Entretanto, as informações disponíveis em relação às causas subjacentes desses sintomas persistentes são limitadas. ^1^

O teste de esforço cardiopulmonar (TECP) é uma ferramenta diagnóstica bem estabelecida para avaliar a etiologia dos sintomas e mecanismos subjacentes que limitam o exercício em doenças cardiovasculares e pulmonares. ^10^ Um estudo inicial com avaliação por TECP em sujeitos pós-COVID-19 identificou uma redução no pico de consumo de oxigênio (VO _2_ ), alcançando 66,2 ± 10,5% dos valores previstos 1 mês após a alta, apesar de a espirometria estar dentro da faixa normal. Além disso, observou-se uma redução do pulso de oxigênio em 70% dos sujeitos, enquanto 80% tinham valores normais de equivalente ventilatório de dióxido de carbono (VE/VCO _2_ ), reforçando a hipótese de que fatores extrapulmonares, tais como o efeito do “repouso no leito” durante a hospitalização, como a possível etiologia das limitações para exercícios. ^11^ Da mesma forma, um estudo com dezoito sujeitos relatou uma redução de 30% do pico de VO _2_ , mas um aumento de VE/VCO _2_ , possivelmente devido ao aumento da quimiossensibilidade e à redução do teor e da extração de oxigênio, como principais contribuintes para a redução da capacidade de exercício. ^12^ Raman et al. ^5^ avaliaram cinquenta e um sujeitos após a alta hospitalar e identificaram uma redução no pico de VO _2_ e na inclinação da eficiência do consumo de oxigênio (OUES), bem como um aumento de VE/VCO _2_ e término do TECP devido principalmente a dor muscular generalizada e fadiga e não a falta de ar, o que sugere descondicionamento e deficiências musculoesqueléticas como a causa provável da limitação para o exercício. Clavario et al. ^13^ também relataram limitações funcionais, especialmente devido a deficiência muscular, em um terço dos sujeitos no pós-COVID-19 3 meses após a alta hospitalar.

Entretanto, todos os estudos anteriores que examinaram resultados relacionados ao TECP no pós-COVID-19 ^5 , 11 , 12^ foram realizados após a alta hospitalar em pacientes com os níveis mais graves da doença. Portanto, o impacto da COVID-19 no TECP nas formas leve a moderada da doença é limitado. Por esse motivo, é necessário ter um melhor entendimento das limitações para o exercício em pacientes ambulatoriais pós-COVID-19 com um perfil clínico mais amplo.

Portanto, nosso objetivo principal foi caracterizar as anormalidades do TECP em subgrupos de sujeitos de níveis diferentes de gravidade de doença no pós-COVID-19 com um espectro clínico amplo e compará-los a um grupo controle. O objetivo secundário foi comparar as variáveis do TECP no pós-COVID-19 aos resultados obtidos antes da infecção para entender melhor os efeitos da COVID-19 na tolerância ao exercício.

## Métodos

### Participantes

Realizamos um estudo de coorte retrospectivo de pacientes ambulatoriais encaminhados à avaliação por TECP em um laboratório experiente na região Centro-Oeste do Brasil de junho de 2020 a agosto de 2021. Os critérios de inclusão foram um histórico clínico de COVID-19 sintomática, confirmada por transcrição reversa seguida da reação em cadeia da polimerase em tempo real, ausência de doença cardiovascular ou pulmonar prévia, e encaminhamento para TECP devido a sintomas persistentes ou para excluir disfunção cardiopulmonar na doença após a fase aguda. Foi selecionado um grupo de controle por pareamento por escores de propensão entre os testes realizados em indivíduos saudáveis, sem doenças cardiopulmonares anteriores e sem COVID-19 durante o mesmo período para garantir restrições semelhantes devido às estratégias de mitigação. Além disso, para a comparação pareada (antes e depois da COVID-19), foram pesquisados TECPs anteriores no banco de dados do laboratório de janeiro de 2011 até fevereiro de 2020, com apenas o TECP mais recente considerado, se mais de um exame fosse encontrado. Todos os exames foram realizados pelo mesmo cardiologista, certificado pela Sociedade Brasileira de Cardiologia. A aprovação do comitê de ética institucional foi obtida (CAAE: 35706720.4.0000.8093) em 16 de setembro de 2021. O termo de consentimento informado foi dispensado, já que os dados foram coletados retrospectivamente.

### Avaliação clínica

Foram realizadas avaliações médicas antes do TECP e foram obtidas informações clínicas relacionadas a comorbidades (fatores de risco cardiovascular e doenças cardiopulmonares anteriores), medicamentos e características demográficas. Informações clínicas relacionadas à COVID-19 também foram coletadas, ou seja, data do aparecimento dos sintomas, manifestações durante a doença viral aguda, imagens de tomografia computadorizada (TC) realizadas e instalação de tratamento utilizada nesse estudo.

### Critérios de gravidade da doença COVID-19

A gravidade da COVID-19 foi classificada de acordo com características clínicas e de imagem: *leve* - indivíduos de qualquer idade com qualquer um dos vários sinais e sintomas da COVID-19, mas que não apresentem falta de ar, dispneia, imagem torácica anormal ou saturação de oxigênio reduzida; *moderada* - indivíduos que apresentam evidência de doença respiratória do trato inferior durante a avaliação clínica ou a obtenção de imagens e que tenham saturação de oxigênio acima de 94% em ar ambiente; *grave* - indivíduos que tenham saturação de oxigênio abaixo de 94% em ar ambiente, frequência respiratória > 30 respirações/min, ou infiltrados pulmonares > 50%; e *crítica* - indivíduos que tenha insuficiência respiratória, choque séptico ou disfunção múltipla de órgãos. Além da doença pulmonar, pacientes com a forma crítica da doença também podem ter sofrido doença cardíaca, hepática, renal, do sistema nervoso central ou trombótica. ^1^

### Teste de esforço cardiopulmonar

O TECP foi realizado em uma esteira (Centurium 200) com análise de gases respiração a respiração (Metalyzer 3B, Cortex). Foi utilizado um protocolo teste de esforço máximo limitado pelos sintomas com rampa individualizada para produzir uma duração de exercício limitada pela fadiga de 8 a 12 min. ^10 , 14^ Precauções para mitigar a transmissão viral foram adotadas seguindo recomendações nacionais. ^15^

As seguintes variáveis de TECP foram obtidas:

- espirometria pré-esforço: volume expiratório forçado em um segundo (VEF1) e capacidade vital forçada (CVF);
- sinais e sintomas clínicos, monitoramento eletrocardiográfico e oximetria de pulso;
- pico de frequência cardíaca (FC, bpm) e FC nos limiares ventilatórios (LV) 1 e 2;
- consumo de oxigênio pico (VO _2_ pico, L/min e mL/kg/min);
- percentual atingido do VO _2_ pico predito (VO _2_ %);
- VO _2_ (mL/kg/min) no LV1 e no LV2;
- pico de pulso de oxigênio (mL/batimento);
- pico de razão de troca respiratória;
- cargas de pico alcançadas (velocidade, km/h, e inclinação, %);
- pico de ventilação por minuto (VE, L/min e prevista por porcentagem) e razão entre VE e ventilação voluntária máxima (VE/VVM);
- pico de VE/VO _2_ ;
- inclinação de equivalente ventilatório de dióxido de carbono (VE/VCO _2_ slope);
- inclinação da eficiência do consumo de oxigênio (OUES).

O pico de VO _2_ e a ventilação de minuto foram expressos como a maior média de 30 segundos obtida no minuto final de esforço. Os valores mais altos obtidos no pico do esforço foram considerados para outras variáveis de pico. O LV1 e o LV2 foram determinados por um médico experiente, e o VE/VCO _2_ slope foi calculado até o LV2. A FC prevista foi calculada pela fórmula 220 - idade (anos) ^16^ e o VO _2_ pico previsto, de acordo com os valores de referência do Centro-Oeste do Brasi. ^17 , 18^ A aptidão cardiorrespiratória (ACR) foi classificada de acordo com a distribuição de percentis do VO _2_ pico nos valores de referência do Centro-Oeste do Brasil, ^19^ que identificaram redução de ACR se os valores medidos estivessem abaixo do 5º percentil. O pico de VE previsto por porcentagem foi calculado de acordo com uma equação de previsão. ^20^ A VVM foi calculada conforme medida ou prevista pelo VEF1 x 40. Os valores de referência brasileiros foram usados para espirometria. ^21^

A etiologia das limitações para o exercício foi identificada pela interpretação clínica das variáveis do TECP de acordo com as análises de gráfico de 9 painéis de Wasserman e os critérios da Sociedade Respiratória Europeia, bem como com os sinais e sintomas do paciente. ^10 , 22 - 24^

Na amostra com TECP antes da infecção disponível, é possível haver avaliações com um intervalo entre antes e depois mais longo. Portanto, para verificar as influências do envelhecimento de VO _2_ pico pós-COVID, os cálculos das diferenças nos valores previstos relacionados à idade nos dois TECPs foram realizados de acordo com os valores de referência brasileiros. ^18^

### Análise estatística

Variáveis categóricas foram descritas usando-se frequências absolutas e relativas e variáveis contínuas não foram distribuídas normalmente, sendo descritas por mediana e intervalo interquartil. A normalidade dos dados foi examinada pelo teste de Shapiro-Wilk. Os testes dos postos sinalizados de Wilcoxon foram usados para comparar variáveis contínuas pareadas (dentro do grupo). Testes de Mann-Whitney ou Kruskal-Wallis com Muller Dunn post hoc compararam variáveis de subgrupo conforme apropriado. A diferença de mediana e o intervalos de confiança (IC) 95% foram calculados pelas estimativas de Hodges-Lehmann. Testes qui-quadrado examinaram variáveis categóricas. O pareamento por escores de propensão foi usado para o grupo de controle saudável pareado com os sujeitos com COVID-19. Os escores de propensão foram estimados de acordo com preditores: sexo, idade, peso, altura e índice de massa corporal (IMC). Uma seleção de pares correspondentes individual, usando a correspondência do mais próximo, foi realizada com base nos escores de propensão estimados de cada sujeito, e a tolerância de correspondência (calibre) foi definida em 0,10. Regressão logística múltipla foi realizada para analisar a capacidade de variáveis independentes, como idade, sexo, gravidade da COVID-19 e presença de obesidade, para prever a redução da ACR. Os riscos relativos (RR) e IC 95% foram calculados. Um p-valor bilateral de <0,05 foi considerado significativo para todas as análises. A análise estatística foi realizada utilizando-se o software IBM-SPSS 28.0.

## Resultados

### Amostra do estudo

**Figura 1:**
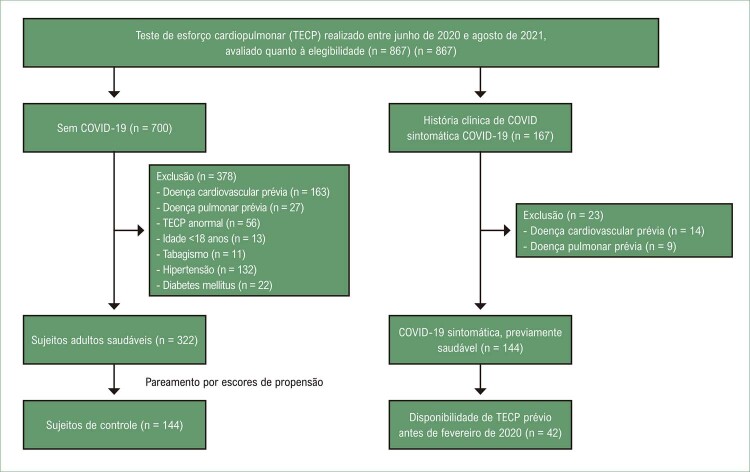
– Fluxograma dos pacientes.

Durante o período do estudo, foram realizadas 867 TECP ( Figura 1 ), e um histórico clínico de COVID-19 sintomática foi identificado em 167 sujeitos. Após excluir sujeitos com doenças cardiovasculares ou pulmonares prévias, uma amostra de 144 sujeitos foi incluída no estudo [idade 43 (36, 53) anos, 57% do sexo masculino] ( Tabela 1 ). As gravidades da doença foram classificadas como leve em 87 (60%), moderada em 30 (21%), grave em 25 (17%), e crítica em 2 (1%) sujeitos. Os dois sujeitos com a forma crítica da doença foram acrescentados ao subgrupo de forma grave para a análise do grupo. O subgrupo de controle (n = 144) foi selecionado por pareamento por escores de propensão de 322 adultos saudáveis sem COVID-19 ( Figura 1 ).

Teste de esforço cardiopulmonar (TECP) realizado entre junho de 2020 e agosto de 2021, avaliado quanto à elegibilidade (n = 867) (n = 867)

**Tabela 1:** – Características demográficas, comorbidades e medicamentos anteriores em sujeitos dos grupos de controle e da COVID-19 por gravidade da doença

Características	Sujeitos de controle	Sujeitos com COVID-19				p valor*
		Geral	Leve	Moderada	Grave	
Sujeitos	144 (100)	144 (100)	87 (60)	30 (21)	27 (19)	
**Dados demográficos**						
Masculino	83 (58)	82 (57)	48 (55)	16 (53)	18 (67)	0,722
Feminino	61 (42)	62 (43)	39 (45)	14 (47)	9 (33)	
Idade, anos	43,0 (38,0, 51,8)	43,0 (36,0, 53,0)	40,0 (33,0, 49,0)	46,5 (40,8, 53,0)	54,0 (44,0, 61,0)	<0,001
Peso, kg	76,3 (65,7, 86,7)	77,2 (67,1, 85,7)	73,1 (65,0, 83,0)	84,4 (69,1, 95,3)	81,1 (73,1, 88,1)	0,026
Altura, m	1,71 (1,63, 1,78)	1,70 (1,63, 1,78)	1,70 (1,61, 1,76)	1,70 (1,63, 1,78)	1,74 (1,66, 1,78)	0,68
IMC, kg/m ^2^	25,9 (23,5, 29,2)	26,0 (23,7, 28,6)	25,0 (23,1, 27,9)	27,7 (25,4, 30,3)	28,3 (26,1, 29,4)	<0,001
Hipertensão	0 (0)	21 (15)	10 (12)	4 (13)	7 (26)	<0,001
Diabetes mellitus	0 (0)	2 (1)	0 (0)	0 (0)	2 (7)	<0,001
**Informações sobre a fase aguda da COVID-19**						
SpO _2_ <94% em ar ambiente		22 (15)	0 (0)	0 (0)	22 (82)	<0,001
Infiltrados pulmonares na TC						
≥50%		18 (13)	0 (0)	0 (0)	18 (67)	<0,001
25-49%		21 (15)	0 (0)	15 (50,0)	6 (22)	
≤24%		17 (12)	0 (0)	15 (50,0)	2 (7)	
Normal		25 (17)	25 (29)	0 (0)	0 (0)	
Não realizado		63 (44)	62 (71)	0 (0)	1 (4)	
Hospitalização		22 (15)	0 (0)	1 (3)	21 (78)	<0,001
Ventilação mecânica		2 (1)	0 (0)	0 (0)	2 (7)	0,012
Sintomas persistentes relacionados à COVID-19		60 (42)	23 (26)	14 (47)	23 (85)	<0,001

Valores expressos como mediana e faixa interquartil ou frequências absolutas e relativas, conforme apropriado. Análise estatística: Comparação entre sujeitos dos grupos de controle e dos grupos com COVID-19 em geral: Teste Mann-Whitney: todas as variáveis demográficas não foram diferentes (pareamento efetivo). * Comparação entre subgrupos de sujeitos de diferentes níveis de gravidade da doença e de controle: Teste de Kruskal-Wallis para variáveis contínuas ou teste Qui-quadrado para variáveis categóricas. IMC: índice de massa corporal; TC: tomografia computadorizada; SpO _2_ : saturação de oxigênio.

A maioria dos pacientes era saudável previamente, com uma frequência baixa de hipertensão ( Tabela 1 ). Durante a fase aguda da COVID-19, o índice de hospitalização foi baixo, com apenas 2 pacientes precisando de ventilação mecânica, embora alguns pacientes tivessem dessaturação de oxigênio em repouso e anormalidades na TC pulmonar. Sintomas residuais relacionados à síndrome do pós-COVID-19 (tais como fadiga, falta de ar, dispneia e tolerância reduzida ao exercício) foram identificados em 60 sujeitos (42%), com aumento da frequência de acordo com a gravidade da doença ( Tabela 1 ).

Sujeitos pós-COVID-19 e de controle não apresentaram diferenças em idade, sexo, dados antropométricos, indicando uma estratégia de pareamento eficaz. Entretanto, ao comparar os grupos com diferentes gravidades de doença e de controle, os sujeitos nos grupos com as formas de doença moderada e grave eram mais velhos e tinham peso e IMC mais altos do que os sujeitos dos grupos de doença leve e de controle ( Tabela 1 ). Entre os subgrupos de pós-COVID-19, a prevalência mais baixa de hipertensão e uso de medicamento foi observada em sujeitos com a forma leve da doença do naqueles com formas de maior gravidade durante a fase aguda.

### Teste de esforço cardiopulmonar

**Figura 2:**
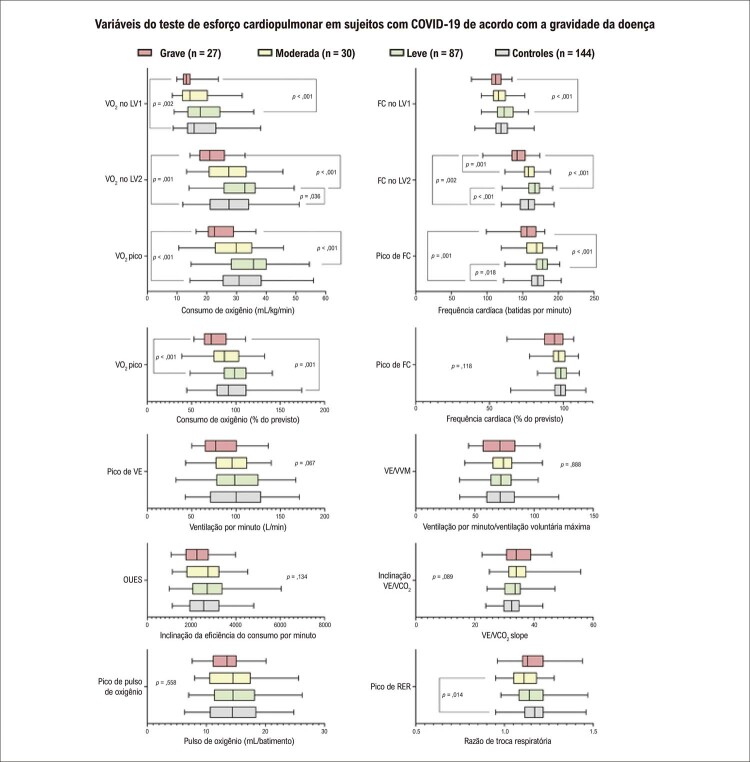
– Variáveis do teste de esforço cardiopulmonar (TECP) em sujeitos com COVID-19 de acordo com a gravidade da doença e os sujeitos do grupo de controle.

O TECP foi realizado em média 11,5 (7,0, 21,2) semanas após a doença aguda com a maioria dos TECP limitados por fadiga do músculo periférico (92%), com limitação mínima devida aos sistemas pulmonar e cardiovascular (6% e 2%, respectivamente) ( Tabela 2 ). Embora a fadiga muscular periférica seja a etiologia predominante da limitação para o exercício, a magnitude da redução da ACR variou entre os grupos de gravidade de COVID-19 diferentes e foi mais baixa nos subgrupos com as formas moderada e grave do que nos sujeitos nos subgrupos de forma leve e de controle. Um VO _2_ % significativamente mais baixo foi observado no subgrupo com a forma grave da doença quando comparado aos subgrupos de forma leve e de controle ( Figura 2 ).

**Tabela 2:** – Variáveis do teste de esforço cardiopulmonar em sujeitos com COVID-19 de acordo com a gravidade da doença e os sujeitos do grupo de controle

Características	Sujeitos de controle (n=144)		Sujeitos com COVID-19				p valor*
			Geral (n=144)	Leve (n=87)	Moderada (n=30)	Grave (n=27)	
**Variáveis de TECP**							
Tempo após a doença aguda da COVID-19, semanas	--	11,5 (7,0, 21,2)		11,4 (6,7, 22,3)	10,5 (6,5, 15,6)	13,6 (7,9, 23,3)	0,288
Duração do exercício, minutos	10,0 (8,9, 1,3)	10,4 (9,0, 1,3)		10,2 (9,0, 11,4)	10,3 (8,8, 11,3)	10,7 (9,4, 11,7)	0,551
Pico de FC, bpm	171 (162, 180)	172 (160, 180)		178 (169, 185)	170 (155, 179)	156 (147, 169)	<0,001 † 0,001; ‡ <0,001; ‖ 0,018
Pico de FC, % do previsto	98,3 (94,0, 101,7)	97,2 (93,2, 101,7)		98,3 (94,5, 102,3)	96,7 (93,1, 101,3)	94,0 (87,0, 100,0)	0,118
Pico de velocidade, km/h	9,5 (7,6, 11,9)	9,2 (7,3, 11,5)		10,6 (8,5, 12,3)	9,3 (6,9, 11,7)	7,1 (6,6, 7,9)	<0,001 † ‡ <0,001; § 0,024
Pico de inclinação, %	3,5 (3,0, 4,0)	3,5 (3,0, 4,0)		3,5 (3,0, 4,0)	3,5 (3,0, 4,0)	4,5 (3,5, 6,0)	<0,001 † 0,001; ‡ <0,001; § 0,005
Pico de RER	1,17 (1,11, 1,22)	1,13 (1,08, 1,21)		1,14 (1,08, 1,22)	1,11 (1,05, 1,18)	1,13 (1,10, 1,22)	0,017 ¶ 0,014
VO _2_ pico, L/min	2,49 (1,76, 3,11)	2,40 (1,85, 2,98)		2,60 (1,95, 3,16)	2,46 (1,67, 3,03)	2,06 (1,48, 2,57)	0,026 0,016
VO _2_ pico, mL/kg/min	30,9 (25,3, 38,4)	31,1 (23,6, 37,5)		35,7 (28,1, 40,1)	29,9 (22,7, 35,3)	22,6 (20,4, 29,1)	<0,001 † ‡ <0,001
VO _2_ pico, % do previsto	91,6 (78,2, 111,8)	92,4 (76,7, 107,4)		98,5 (86,5, 111,8)	87,1 (74,9, 103,6)	72,2 (64,1, 89,4)	<0,001 † 0,001; ‡ <0,001;
Pico de pulso de oxigênio, mL/batimento	14,4 (10,6, 18,4)	14,0 (11,1, 17,4)		14,5 (11,3, 18,2)	14,5 (10,5, 17,5)	13,5 (11,1, 15,1)	0,558
OUES	2.544 (1.900, 3.246)	2.570 (1.968, 3.236)		2.707 (2.035, 3.389)	2.742 (1.767, 3.248)	2.240 (1.729, 2.767)	0,134
Pico de VE, L/min	100,1 (70,9, 128,1)	94,8 (75,6, 118,8)		98,5 (77,9, 125,2)	95,5 (77,1, 112,5)	77,0 (64,9, 100,9)	0,067
Pico de VE, % do previsto	106,2 (92,4, 118,7)	104,9 (93,6, 115,5)		104,9 (96,0, 116,6)	108,8 (91,8, 115,6)	94,3 (75,1, 111,7)	0,103
Pico de VE/VVM, %	71,3 (59,7, 83,7)	72,0 (63,1, 81,1)		71,9 (63,3, 80,9)	74,2 (65,0, 81,5)	71,1 (56,6, 84,1)	0,888
Pico de VE/VO _2_	40,2 (37,0, 43,3)	40,2 (36,4, 44,0)		39,8 (36,1, 44,0)	40,4 (36,9, 42,6)	42,1 (37,4, 46,2)	0,503
VE/VCO _2_ slope	32,5 (29,7, 35,1)	33,8 (30,6, 36,4)		33,6 (30,0, 35,5)	34,0 (31,4, 37,4)	34,0 (30,6, 39,0)	0,089
VO _2_ no LV1, mL/kg/min	15,8 (13,4, 23,2)	15,6 (12,9, 23,1)		17,8 (13,5, 24,5)	14,3 (11,7, 20,3)	13,1 (12,0, 14,5)	<0,001 † 0,002; ‡ <0,001;
FC no LV1, bpm	120 (112, 129)	120 (112, 130)		124 (114, 137)	116 (109, 126)	112 (106, 120)	<0,001 ‡ <0,001
VO _2_ no LV2, mL/kg/min	27,5 (21,0, 34,2)	28,5 (22,3, 35,0)		32,8 (25,7, 36,4)	27,4 (20,7, 33,4)	21,0 (17,5, 26,1)	<0,001 † 0,001; ‡ <0,001; ‖ 0,036
FC no LV2, bpm	158 (146, 167)	162 (151, 172)		167 (158, 174)	158 (152, 167)	142 (135, 154)	<0,001 † 0,002; ‡ ‖ <0,001; § 0,011
Redução de SpO _2_ no exercício	0 (0)	7 (4,9)		0 (0)	0 (0)	7 (25,9)	<0,001
**Motivo para término do TECP**							
Fadiga muscular	144 (100)	132 (92)		85 (98)	28 (93)	19 (70)	<0,001
Limitação cardiovascular	0 (0)	3 (2)		2 (2)	0 (0)	1 (4)	
Limitação pulmonar	0 (0)	9 (6)		0 (0)	2 (7)	7 (26)	
Total	144 (100)	144 (100)		87 (100)	30 (100)	27 (100)	

Valores expressos como mediana e intervalo interquartil ou frequências absolutas e relativas. * Comparação entre subgrupos de sujeitos de diferentes níveis de gravidade da doença e de controle: Teste de Kruskal-Wallis com teste Muller Dunn post hoc para variáveis contínuas ou teste Qui-quadrado para variáveis categóricas. P valores pós-teste relatados quando o p valor de Kruskal-Wallis <0,05: † Grave versus Controle; ‡ Grave versus Leve; § Grave versus Moderada; ‖Leve versus Controle; ¶ Moderada versus Controle; # Moderada versus Leve. TECP: teste de esforço cardiopulmonar; FC: frequência cardíaca; VVM: ventilação voluntária máxima; OUES: inclinação da eficiência do consumo de oxigênio; RER: razão de troca respiratória; SpO_ 2: saturação de oxigênio; VCO2: produção de dióxido de carbono; VE: ventilação por minuto; VO2: consumo de oxigênio; LV: limiar ventilatório_.

Sujeitos com TECP após a COVID-19 (n = 144) e de controle (n = 144). Valores expressos como mediana, intervalo interquartil, e limites. FC: frequência cardíaca; OUES: inclinação da eficiência do consumo de oxigênio; RER: razão de troca respiratória, VCO _2_ : produção de dióxido de carbono; VE: ventilação por minuto; VO _2_ : consumo de oxigênio; LV: limiar ventilatório.

**Figura 3:**
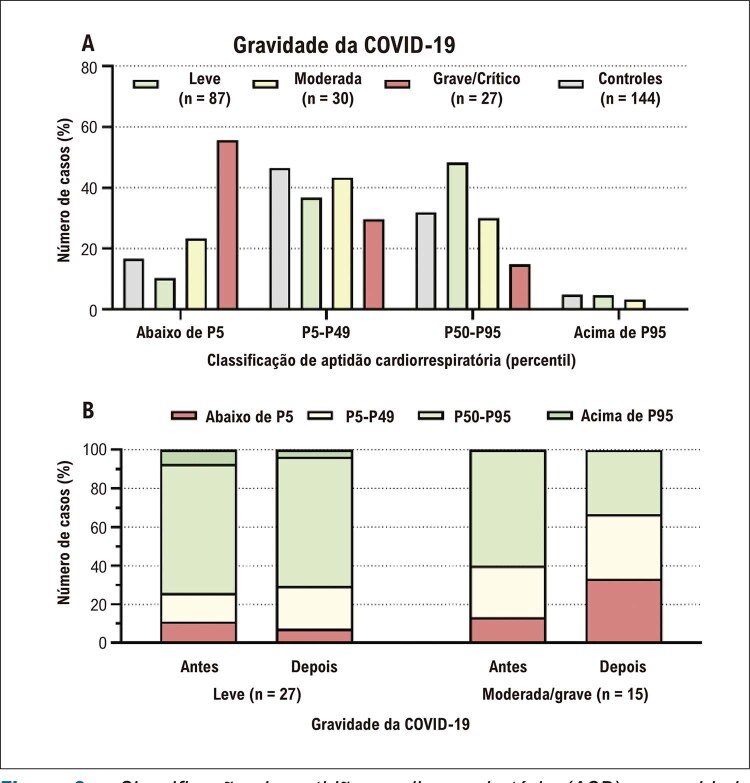
– Classificação da aptidão cardiorrespiratória (ACR) e gravidade da COVID-19. A) Dados para sujeitos com teste de esforço cardiopulmonar (TECP) após a COVID-19 (n = 144) e controles (n = 144); B) Dados para um subgrupo com TECP antes e depois da doença viral (n = 42). Os valores de gravidade da doença apresentados como porcentagem de distribuição (%) e a ACR, como percentil de classificação, de acordo com o pico de consumo de oxigênio.

Sujeitos dos subgrupos de gravidade moderada exibiram uma mediana de VO _2_ pico 5,8 mL/kg/min mais baixa que os do subgrupo de doença leve, e 1,0 mL/kg/min mais baixos do que os do subgrupo de controle, embora as diferenças não sejam significativas. Sujeitos no subgrupo de doença grave apresentaram limitações maiores e uma mediana de VO _2_ pico mais baixa quando comparada ao subgrupo com a doença leve e o subgrupo de controle (13,1 e 8,3 mL/kg/min, respectivamente) ( Tabela 2 e Figura 2 ). Como resultado, a classificação de ACR foi significativamente diferente entre os grupos de gravidade diferente ( Figura 3A ), com ACR reduzida em 56% dos sujeitos com as formas graves da doença e frequências mais baixas em grupos com formas moderada e leve da doença (23% e 10%, respectivamente).

De acordo com a regressão logística múltipla, idade e sexo não puderem prever uma ACR reduzida, enquanto a gravidade da COVID-19 e obesidade puderam (r2 = 0,46). Sujeitos com a forma grave da COVID-19 tinham um risco mais alto de ter ACR reduzida [RR: 8,6 (IC 95%: 2,9 a 25,7)] comparados com os controles. Diferentemente, as gravidades de doença leve ou moderada não foram associadas a uma ACR mais baixa. Da mesma forma, as classificações de obeso e sobrepeso foram identificadas como um preditor robusto de ACR reduzida [RR: 37,4 (IC 95%: 11,7 a 119,9) e [RR: 3,4 (IC de 95% 1,07 a 11,1), comparado com sujeitos com pesos saudáveis.

Em relação aos limiares ventilatórios, níveis de VO _2_ significativamente mais baixos no LV1 e no LV2 também foram encontrados em sujeitos com formas mais graves da doença ( Tabela 2 e Figura 2 ). Da mesma forma, a FC no pico e nos LV apresentou semelhanças, com valores significativamente mais baixos no grupo com doença grave. O pico de ventilação minuto, a OUES, e o pico de pulso de oxigênio revelaram valores medianos que eram mais baixos nos sujeitos com a forma grave da doença, mas as diferenças entre os outros subgrupos e controles não foram estatisticamente significativas. Nenhuma das demais variáveis de TECP foi significativamente diferente entre os subgrupos de diferentes gravidades da doença COVID-19 ( Tabela 2 e Figura 2 ).

A espirometria em repouso realizada antes do TECP identificou valores normais em vários sujeitos no pós-COVID-19 com anormalidades leves em apenas 13% dos sujeitos com base nos valores de CVF e em 14% com base nos valores de VEF1. Embora não sejam estatisticamente significativos, sujeitos com a forma grave da doença que apresentam uma proporção mais alta de anormalidades leves com base nos valores de CVF e de VEF1 (29% e 18%, respectivamente). Entretanto, os percentuais dos valores preditos foram significativamente mais baixos em sujeitos com a forma grave da doença do que nos controles (CVF: 85,7 versus 99%, p = 0,022 e VEF1: 85,5 versus 94,3%, p = 0,007).

### Subgrupo de pacientes com avaliação por TECP anterior

Quarenta e dois dos 144 sujeitos (29%) tinham um TECP anterior disponível para comparação ( Figura 1 ). Esse subgrupo de sujeitos teve a gravidade da COVID-19 classificada como leve em 27 sujeitos (64%), moderada em 9 (21%), e grave em 6 (14%). Para a análise dos grupos com gravidade diferentes, os grupos de gravidade moderada e grave foram combinados.As características desses 42 sujeitos foram semelhantes às de toda a amostra do estudo ( Tabela 3 ). Durante a fase aguda da COVID-19, o índice de hospitalização também foi baixo, e a ventilação mecânica não foi necessária para nenhum sujeito.

**Tabela 3:** – Dados demográficos, medicamentos anteriores, e informações clínicas sobre COVID-19 em um subgrupo de sujeitos com avaliação prévia

Características	Sujeitos com COVID-19			p Valor *
	Geral	Leve	Moderada/grave	
**Sujeitos**	**42 (100)**	**27 (64)**	**15 (36)**
**Dados demográficos**				
Masculino	27 (64)	17 (63)	10 (67)	0,810
Feminino	15 (36)	10 (37)	5 (33)	
Idade, anos	46,5 (40,0, 54,0)	43,0 (36,0, 51,0)	52,0 (44,0, 55,0)	0,030
Peso, kg	81,4 (70,4, 85,2)	82,0 (69,2, 85,2)	81,3 (73,3, 87,0)	0,906
Altura, cm	173,5 (165,0, 178,0)	175,0 (168,0, 180,0)	173,0 (164,0, 178,0)	0,264
IMC, kg/m ^2^	25,8 (23,4, 27,5)	25,2 (23,0, 27,3)	26,4 (24,0, 27,8)	0,259
Hipertensão	8 (19)	6 (22)	2 (13)	0,482
Diabetes mellitus	0 (0)	0 (0)	0 (0)	--
Informações sobre a fase aguda da COVID-19				
SpO _2_ <94% em ar ambiente	4 (10)	0 (0)	4 (27)	0,005
Hospitalização	4 (10)	0 (0)	4 (27)	0,005
Sintomas persistentes relacionados à COVID-19	14 (33)	6 (22)	8 (53)	0,040

Valores expressos como mediana e faixa interquartil ou frequências absolutas e relativas. Análise estatística: * Comparação entre subgrupos de níveis de gravidade da doença: Teste de Mann-Whitney para variáveis contínuas ou teste Qui-quadrado para variáveis categóricas. IMC: índice de massa corporal; SpO _2_ : saturação de oxigênio.

O TECP antes da infecção por COVID-19 foi realizado com um intervalo mediano de 25,0 (16,8, 40,0) e 39,4 (19,9, 67,5) meses nos subgrupos de forma da doença leve e moderada/grave da doença, respectivamente, e essa diferença não foi significativa.

**Figura 4:**
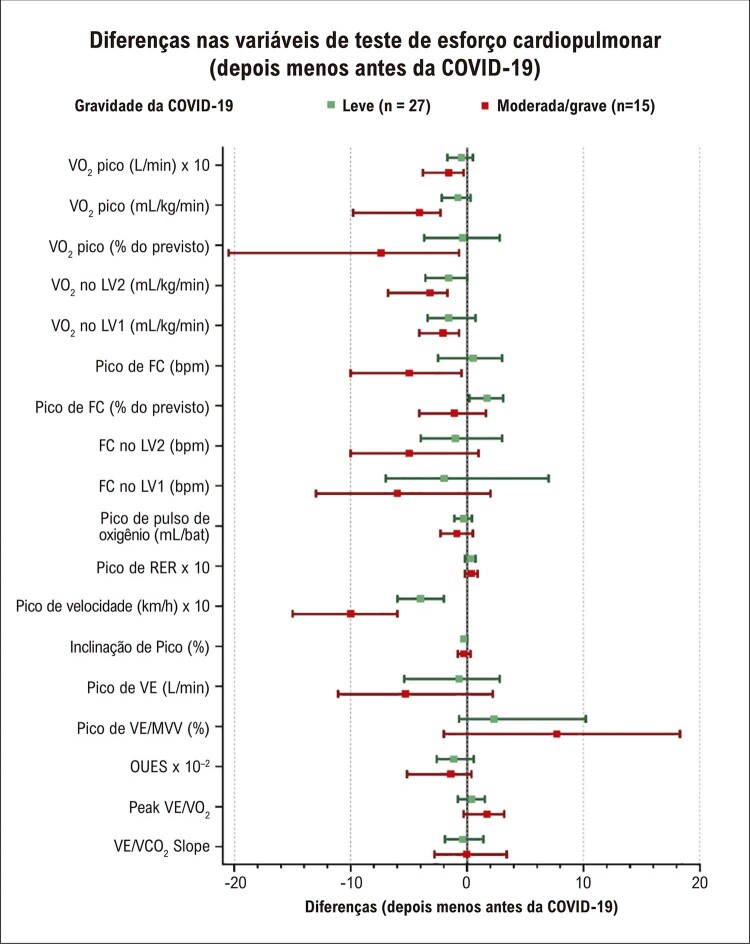
– Diferenças de variáveis em testes de esforço cardiopulmonar (TECP) de acordo com a gravidade da COVID-19. Subgrupo de pacientes (n = 42) com TECP antes e depois da COVID-19. Os valores são diferença de mediana e o IC 95%. FC: frequência cardíaca; OUES: inclinação da eficiência do consumo de oxigênio; RER: razão de troca respiratória; VCO 2 : produção de dióxido de carbono; VE: ventilação por minuto; VO 2 : consumo de oxigênio; LV: limiar ventilatório. Algumas variáveis foram ajustadas para uma escala gráfica. VO 2 pico (L/min), pico de RER, e pico de velocidade (km/h), multiplicando por 10; OUES, multiplicando por 10 -2 .

No subgrupo com a forma leve da doença de 27 sujeitos, o TECP antes e depois da infecção de COVID-19 não revelou mudanças significativas na maioria das medidas, exceto por uma redução mediana na velocidade de pico da esteira de 0,4 km/h [IC 95%: -0,6 a -0,2], uma pequena redução na duração do exercício de 0,5 minutos, e um pequeno aumento no percentual atingido do pico do FC ( Tabela 4 e Figura 4 ).

**Tabela 4:** – Variáveis de teste de esforço cardiopulmonar no subgrupo com exame anterior e divisão de acordo com a gravidade da COVID-19

	Gravidade da COVID-19					
	Leve (n = 27)			Moderada ou grave (n = 15)		
Variáveis de TECP	Antes	Depois	p Valor * Diferença †	Antes	Depois	p Valor * Diferença †
Duração do exercício, minutos	10,6 (9,9, 11,3)	10,4 (9,0, 11,1)	-0,5 (-1,0 a -0,05) p = 0,037	11,3 (9,9, 12,4)	10,4 (9,0, 11,3)	-1,0 (-1,8 a -0,2) p = 0,022
Pico de FC, bpm	173 (163, 181)	173 (160, 185)	0,5 (-2,5 a 3,0) p = 0,829	173 (168, 178)	169 (156, 176)	-5 (-10 a -0,5) p = 0,029
Pico de FC, % do previsto	96,3 (93,5, 99,4)	97,3 (94,4, 102,3)	1,7 (0,2 a 3,1) p = 0,027	97,8 96,1 (102,9)	98,1 93,7 (101,7)	-1,1 (-4,1 a 1,6) p = 0,49
Pico de velocidade, km/h	11,9 (11,0, 14,1)	11,7 (10,6, 14,2)	-0,4 (-0,6 a -0,2) p = 0,005	11,2 (8,2, 13,2)	9,5 (6,8, 12,5)	-1,0 (-1,5 a -0,6) p = 0,001
Pico de inclinação, %	3,5 (3,0, 3,5)	3,5 (3,0, 3,5)	-0,3 (-0,5 a 0) p = 0,048	4,0 (3,5, 4,0)	3,5 (3,0, 4,0)	-0,3 (-0,8 a 0,3) p = 0,259
Pico de RER	1,10 (1,06, 1,21)	1,14 (1,08, 1,22)	0,03 (-0,02 a 0,07) p = 0,218	1,13 (1,11, 1,17)	1,17 (1,10, 1,24)	0,04 (-0,02 a 0,09) p = 0,196
VO _2_ pico, L/min	2,93 (2,00, 3,72)	3,08 (2,09, 3,48)	-0,05 (-0,17 a 0,05) p = 0,249	2,29 (2,04, 3,28)	2,12 (1,84, 2,96)	-0,16 (-0,38 a -0,03) p = 0,023
VO _2_ pico, mL/kg/min	40,8 (33,7, 44,7)	38,0 (33,4, 41,0)	- 0,8 (-2,2 a 0,3) p = 0,175	34,1 (26,5, 40,5)	27,8 (22,3, 36,0)	-4,1 (-9,8 a -2,3) p < 0,001
VO _2_ pico, % do previsto	110,4 (99,8, 122,1)	106,3 (98,2, 124,8)	-0,4 (-3,7 a 2,8) p = 0,866	108,1 (83,9, 113,6)	90,1 (69,6, 103,9)	-7,4 (-20,5 a -0,7) p = 0,041
Pico de pulso de oxigênio, mL/batimento	17,7 (11,8, 21,6)	17,6 (13,0, 19,8)	-0,3 (-1,1 a 0,4) p = 0,414	14,4 (11,8, 19,1)	13,6 11,1 (16,6)	-0,9 (-2,3 a 0,5) p = 0,256
OUES	3.075 (2.176, 3.706)	2.977 (2.283, 3.554)	-115 -262 a 56 p = 0,121	2.481 (2.209, 3.215)	2.260 (2.086, 3.047)	-140 (-517 a 37) p = 0,078
Pico de VE, L/min	121,3 (89,1, 139,4)	119,2 (90,8, 139,3)	-0,7 (-5,4 a 2,8) p = 0,639	98,4 (80,8, 126,3)	95,3 (78,5, 127,4)	-5,3 (-11,1 a 2,2) p = 0,147
Pico de VE, % do previsto	112,8 (101,9, 120,8)	114,2 (105,1, 125,4)	1,6 (-2,1 a 5,2) p = 0,374	114,2 (95,1, 122,5)	111,1 (95,9, 116,2)	- 2,8 (-10,1 a 6,3) p = 0,570
Pico de VE/VVM, %	74,9 (67,4, 81,7)	77,9 (71,9, 85,7)	2,3 (-0,7 a 10,2) p = 0,097	72,80 (62,8, 79,2)	78,1 (71,2, 89,0)	7,7 (-2,0 a 18,3) p = 0,100
Pico de VE/VO _2_	39,6 (36,9, 42,4)	40,0 (36,5, 42,9)	0,4 (-0,8 a 1,5) p = 0,360	39,1 (37,6, 45,3)	42,4 (40,2, 45,0)	1,7 (-0,3 a 3,2) p = 0,167
VE/VCO _2_ slope	33,9 (31,6, 36,0)	34,2 (30,6, 35,5)	-0,4 (-1,9 a 1,4) p = 0,631	31,4 (29,6, 36,8)	33,1 (31,5, 34,9)	-0,05 (-2,8 a 3,4) p = 0,977
VO _2_ no LV1, mL/kg/min	25,2 (18,8, 28,1)	23,9 (18,5, 26,3)	-1,6 (-3,4 a 0,7) p = 0,133	19,8 (13,7, 25,6)	14,9 (12,8, 23,9)	-2,1 (-4,1 a -0,7) p = 0,011
FC no LV1, bpm	137 (119, 143)	129 (116, 143)	-2 (-7 a 4) p = 0,400	124 (112, 130)	117 (112, 126)	-6 (-13 a 2) p = 0,094
VO _2_ no LV2, mL/kg/min	38,3 (29,6, 42,8)	35,1 (32,7, 37,5)	-1,6 (-3,6 a 0) p = 0,05	30,7 (23,8, 37,4)	27,4 (21,8, 33,1)	-3,2 (-6,8 a -1,7) p = 0,003
FC no LV2, bpm	164 (158, 174)	166 (151, 174)	-1 (-4 a 3) p = 0,637	159 (156, 165)	156 (146, 165)	-5 (-10 a 1) p = 0,069

Valores expressos como mediana e intervalo interquartil. Estatísticas: * O teste dos postos sinalizados de Wilcoxon foi usado para comparar variáveis antes e depois dentro de cada subgrupo de gravidade de COVID-19. † Diferenças de mediana (depois menos antes) e estimativas de IC 95% calculadas pelo método de Hodges-Lehman. TECP: teste de esforço cardiopulmonar; FC: frequência cardíaca; VVM: ventilação voluntária máxima; RER: razão de troca respiratória; VO2: consumo de oxigênio; VCO2: produção de dióxido de carbono; VE: ventilação por minuto; LV: limiar ventilatório; OUES: inclinação da eficiência do consumo de oxigênio.

No subgrupo com a forma moderada/grave da doença, composto de 15 sujeitos, observou-se uma redução mediana significativa no pico de velocidade, mas com uma magnitude maior [1,0 km/h (IC 95%: -1,5 a -0,6). Além disso, foram observadas reduções significativas no VO _2_ pico e nos LV. A redução mediana no VO _2_ pico de 4,1 mL/kg/min (IC 95%: -9,8 a -2,3) refletiu uma redução de 7,4% (IC 95%: -20,5 a -0,7) no VO _2_ % pico ( Tabela 4 e Figura 4 ). Da mesma forma, um nível reduzido de ACR foi identificado anteriormente em 13% dos sujeitos, que aumentou para 33% após a COVID-19 ( Figura 3B ). Considerando a redução do VO _2_ pico relacionada à idade devido ao intervalo entre as avaliações de TECP, a diferença mediana de valores previstos foi de -0,8 (-0,7 a -1,4) mL/kg/min, que foi responsável por apenas cerca de 20% da redução mediana do VO _2_ pico.

O pico de FC também foi significativamente mais baixo após a COVID-19, com uma diferença mediana de - 5 batimentos por minuto (IC 95%: -10 a -0,5). Apesar das alterações significativas observadas nos VO _2_ pico e FC, não houve diferenças significativas em equivalentes ventilatórios, OUES, pico de pulso de oxigênio, pico de ventilação minuto e outras variáveis de TECP antes e depois da COVID-19 no subgrupo com as formas moderada/grave da doença.

## Discussão

Até onde sabemos, este é um dos maiores estudos a examinar os resultados de TECP em sujeitos no pós-COVID-19, que apresenta um espectro clínico amplo e que comparou resultados de TECP com um grupo de controle selecionado por paramento por escores de propensão. Além disso, a comparação de TECP realizados antes da COVID-19 em quase 30% da coorte do estudo é notável e reforça a relevância, a novidade e a importância dos achados do estudo.

Nossos resultados indicam que pacientes que apresentaram COVID-19 na forma grave têm uma redução significativa na ACR três meses após o aparecimento do sintoma, confirmada por um VO _2_ pico mediano mais baixo do que o de sujeitos com a forma leve da doença e do grupo de controle. A comparação pareada de sujeitos com um TECP anterior à COVID-19 corrobora os achados de toda a coorte. Além do mais, o gráfico de 9 painéis e os sinais e sintomas observados durante o TECP revelam que a fadiga do músculo periférico foi a causa mais prevalente de limitações para os exercícios em sujeitos no pós-COVID-19. Esses achados são semelhantes aos de estudos anteriores, incluindo sujeitos hospitalizados. ^5 , 11 - 13^

Estudos recentes de TECP em sujeitos no pós-COVID-19 encontraram uma redução de VO _2_ pico semelhante de 30 a 34% dos valores previstos. ^5 , 11 , 12^ Fatores extrapulmonares, tais como hospitalização “repouso no leito”, ^11^ anemia, ^12^ e fraqueza muscular, ^5^ foram apresentados como possíveis mecanismos subjacentes para uma ACR reduzida. Nossos resultados corroboram esses fatores extrapulmonares, já que os sujeitos com formas moderada e grave da doença tinham valores de VO _2_ % que eram 11,4% e 26,3% menos que o subgrupo com a forma leve da doença ( Tabela 2 ), destacando o papel que a fadiga do músculo periférico teve na limitação do exercício em nossa coorte composta principalmente de sujeitos não hospitalizados no pós-COVID-19. Além disso, nos 42 pacientes com TECP anterior à COVID-19, foram observadas reduções significativas no VO _2_ pico e no VO _2_ % apenas nos sujeitos com formas mais graves de doenças, o que não poderia ser explicado pela diferença de idade entre as avaliações por TECP. Considerando o que foi disposto acima, nossos achados e os de outros estudos identificaram os efeitos mais danosos que a forma grave da COVID-19 tem na tolerância ao exercício e no desempenho musculoesquelético. ^5 , 12 , 13 , 25^

Semelhante aos estudos anteriores com pacientes hospitalizados, a ACR reduzida devido a deficiência músculo esquelética periférica causada pela extração de oxigênio periférica anormal causada pelo catabolismo do músculo parece ser uma consequência mais provável da COVID-19 do que um efeito do “repouso no leito”. ^12^ Esse mecanismo é fortalecido pelas reduções marcadas observadas no VO _2_ no LV1 e no LV2, comparando grupos com gravidades diferentes de doença e de controle ( Tabela 2 e Figura 2 ), bem como a comparação pareada antes e depois da COVID-19 na maioria dos sujeitos não hospitalizados ( Tabela 4 e Figura 4 ). Essas observações indicam a ativação precoce de metabolismo anaeróbico e menor efeito de tampão durante o exercício, ^10^ que pode ser um dos principais mecanismos responsáveis pelos sintomas de fadiga persistentes em pacientes no pós-COVID-19. Da mesma forma, Singh et al. ^25^ utilizando avaliação por TECP invasiva, relataram uma redução marcada no VO _2_ associada à extração de oxigênio deficiente, apesar de um índice cardíaco preservado, reforçando um limite periférico, e não cardíaco, durante o exercício.

Além do mais, observamos um pico de FC significativamente mais baixo em sujeitos com forma grave da doença comparados aos do grupo de controle e aos do subgrupo de indivíduos com forma leve da doença ( Figura 2 ), bem como um pico de FC mais baixo durante o TECP realizado no pós-COVID-19 em comparação com o TECP anterior à COVID-19 no subgrupo com a forma moderada/grave da doença ( Figura 4 ). Esse achado é corroborado por um estudo anterior em sujeitos com COVID-19 ^12^ que relatou uma redução similar no pico de FC. Também identificamos que a FC no LV1 e no LV2 era significativamente mais baixa no subgrupo com a forma grave da doença em comparação com os subgrupos com a forma leve da doença e de controle, mas essa redução não foi observada na comparação pareada. A combinação de valores de pico de FC e de VO _2_ pico mais baixos e LV precoce pode ser atribuída à miopatia metabólica que limita o exercício, ^22^ o que corrobora a fadiga muscular como a principal causa para a limitação do exercício em nosso estudo. É importante destacar que essas diferenças em valores de pico não foram associadas à intensidade de esforço relativa mais baixa, já que o pico de razão de troca respiratória não era diferente entre os sujeitos dos subgrupos de gravidade de doença diferentes e de controle, nem durante as comparações antes e depois da COVID-19 pareadas.

Apesar das diferenças relatadas em VO _2_ e FC no pico e no LV1 e LV2, nenhuma outra medida de TECP foi significativamente diferente entre os subgrupos de gravidade de doença diferentes e de controle ( Tabela 2 e Figura 2 ) nem durante as comparações antes e depois da COVID-19 pareadas. Apesar do envolvimento pulmonar em sujeitos mais gravemente afetados durante a infecção aguda por COVID-19 e um índice de 42% de sintomas residuais, não encontramos diferenças em eficácia ventilatória, quantificada VE/VCO _2_ slope, nem nas comparações com par correspondente entre o grupo de controle ( Tabela 2 e Figura 2 ), ou na análise pareada com dados anteriores à COVID-19 ( Tabela 4 e Figura 4 ), que são semelhantes aos resultados relatados por Gao et al. ^11^

Entretanto, vários outros estudos ^5 , 12 , 25^ relataram valores maiores de VE/VCO _2_ slope em sujeitos com COVID-19 comparados com os controles, o que, conforme Baratto et al., ^12^ foram atribuídos ao aumento da quimiossensibilidade estimulando uma ventilação mais alta. Outra diferença em relação aos nossos resultados é que foram relatados valores mais baixos de OUES ^5^ e pulso de oxigênio ^11 , 12^ em sujeitos no pós-COVID-19. No entanto, os sujeitos nesses estudos foram avaliados na admissão hospitalar ou logo após ela, com uma possibilidade mais alta de disfunção ou injúria miocárdica, ^8 , 26^ que poderiam ter influenciado os resultados do TECP relatados nesses estudos. Em nosso estudo, pacientes no pós-COVID-19 com um espectro clínico amplo foram avaliados, e apenas 15% dos sujeitos haviam sido hospitalizados anteriormente durante a fase viral aguda. As características da nossa amostra de participantes podem ter produzido resultados diferentes nessas variáveis de TECP.

De acordo com a regressão logística múltipla, identificamos que a COVID-19 grave estava associada a um risco quase 9 vezes maior de apresentar ACR reduzida, destacando o impacto da gravidade da doença na limitação para o exercício. Apesar de idade e sexo não serem preditores, a obesidade foi um preditor robusto, com um risco aumentado em 37 vezes. O impacto do peso e da gravidade da doença na limitação para o exercício também foi demonstrado por Braga et al. ^27^ ; entretanto, nesse estudo, a idade e o sexo foram preditores de ACR reduzida, que é o oposto de nossos achados.

Por último, é essencial destacar que os principais achados do presente estudo indicam que a principal razão para a limitação para o exercício, e provavelmente o motivo dos sintomas persistentes, foi a limitação do músculo periférico na maioria dos sujeitos (92%). Limitações pulmonares e cardiovasculares foram identificadas em apenas 12 sujeitos (8%) que foram afetados mais gravemente. No subgrupo com a forma grave da doença, a principal limitação também foi fadiga do músculo periférico (70%), dos quais 85% relataram sintomas persistentes. Entretanto, 26% dos pacientes nesse subgrupo apresentaram reduções significativas na oximetria de pulso durante o exercício, apesar de a maioria ter espirometria normal. Esse achado reforça a importância de se usar TECP em sujeitos com sintomas persistentes, especialmente nos afetados por formas mais graves da COVID-19, já que a espirometria normal ou quase normal em repouso pode não ser suficiente para excluir a disfunção pulmonar em pacientes no pós-COVID-19.

### Limitações do estudo

Este estudo tem várias limitações que precisam ser descritas. Primeiramente, é um estudo retrospectivo unicêntrico realizado em um laboratório ambulatorial privado, o que resulta em viés de seleção. Além disso, os indivíduos encaminhados à avaliação por TECP podem ser mais sintomáticos do que os indivíduos não encaminhados para o TECP, o que pode fazer com que os resultados do estudo sejam mais aplicáveis a pacientes com COVID longa e, portanto, um ponto forte e não uma limitação de nosso estudo. Ademais, sujeitos com a forma crítica da doença não foram adequadamente representados em nosso estudo e é possível que a redução de ACR fosse ainda mais pronunciada nesse subgrupo. O intervalo entre as avaliações variou na amostra com o TECP anterior à infecção viral. No entanto, os cálculos de reduções relacionadas à idade no VO _2_ pico previsto demonstraram que o envelhecimento poderia explicar aproximadamente 20% da redução observada na variável. Portanto, consideramos que nossos achados estavam principalmente relacionados à COVID-19 e não ao intervalo entre as avaliações. Por último, embora este seja um estudo relativamente de médio prazo (TECP realizado em uma mediana de 11,5 semanas após o aparecimento da doença), é necessário um período de acompanhamento mais longo para entender melhor a COVID longa e os impactos clínicos da intolerância ao exercício, bem como os efeitos que o treinamento de exercício pode ter nessa população de pacientes.

## Conclusões

A fadiga do músculo periférico foi a etiologia de limitação de exercício mais comum em pacientes pós-COVID-19 independentemente da gravidade da doença, e foram observadas reduções principalmente em VO _2_ e FC no pico do exercício e no limiar ventilatório. Os equivalentes ventilatórios, a OUES, e o pico de pulso de oxigênio não variaram de acordo com a gravidade da doença ou nas comparações antes e depois da COVID-19. Nossos dados reforçam a importância de se usar o TECP em sujeitos no pós-COVID-19 com sintomas residuais para ajudar a descobrir os sistemas mais comprometidos para personalizar os esforços de reabilitação.
