# Supplementary material for: Teste Cardiopulmonar em Pacientes Pós-COVID-19: De Onde vem a Intolerância ao Exercício?
Source: Arq Bras Cardiol. 2023 Feb 16;120(2):e20220150. [Article in Portuguese] doi: 10.36660/abc.20220150 (PMC12080623; doi:10.36660/abc.20220150)
Supplement: Translation

# Translation: Cardiopulmonary Exercise Testing in Post-COVID-19 Patients: Where Does Exercise Intolerance Come From?

**Authors:** Mauricio Milani, Juliana Goulart Prata Oliveira Milani, Graziella França Bernardelli Cipriano, Lawrence Patrick Cahalin, Ricardo Stein, Gerson Cipriano

**Affiliations:** 1 Brasil Fitcordis Medicina do Exercício, Brasília, DF – Brasil; 2 Brasil Universidade de Brasília - Programa de Pós-Graduação em Ciências e Tecnologias da Saúde, Brasília, DF – Brasil; 3 Brasil Universidade de Brasília - Programa de Ciências da Reabilitação, Brasília, DF – Brasil; 4 EUA Department of Physical Therapy, University of Miami, Miller School of Medicine, Florida – EUA; 5 Brasil Universidade Federal do Rio Grande do Sul – Programa de Pós-Graduação em Cardiologia e Ciências Cardiovasculares, Porto Alegre, RS – Brasil

**Keywords:** COVID-19, Exercise Test, Cardiorespiratory Fitness

## Abstract

**Background:**

Post-COVID-19 exercise intolerance is poorly understood. Cardiopulmonary exercise testing (CPET) can identify the underlying exercise limitations.

**Objectives:**

To evaluate the source and magnitude of exercise intolerance in post-COVID-19 subjects.

**Methods:**

Cohort study assessing subjects with different COVID-19 illness severities and a control group selected by propensity score matching. In a selected sample with CPET prior to viral infection, before and after comparisons were performed. Level of significance was 5% in the entire analysis.

**Results:**

One hundred forty-four subjects with COVID-19 were assessed (median age: 43.0 years, 57% male), with different illness severities (60% mild, 21% moderate, 19% severe). CPET was performed 11.5 (7.0, 21.2) weeks after disease onset, with exercise limitations being attributed to the peripheral muscle (92%), and the pulmonary (6%), and cardiovascular (2%) systems. Lower median percent-predicted peak oxygen uptake was observed in the severe subgroup (72.2%) as compared to the controls (91.6%). Oxygen uptake differed among illness severities and controls at peak and ventilatory thresholds. Conversely, ventilatory equivalents, oxygen uptake efficiency slope, and peak oxygen pulse were similar. Subgroup analysis of 42 subjects with prior CPET revealed significant reduction in only peak treadmill speed in the mild subgroup and in oxygen uptake at peak and ventilatory thresholds in the moderate/severe subgroup. By contrast, ventilatory equivalents, oxygen uptake efficiency slope, and peak oxygen pulse did not change significantly.

**Conclusions:**

Peripheral muscle fatigue was the most common exercise limitation etiology in post-COVID-19 patients regardless of the illness severity. Data suggest that treatment should emphasize comprehensive rehabilitation programs, including aerobic and muscle strengthening components.

**Central Illustration:** : Cardiopulmonary Exercise Testing in Post-COVID-19 Patients: Where Does Exercise Intolerance Come From?

## Introduction

COVID-19 is a multisystemic disease with acute manifestations ranging from asymptomatic to critical.^1^Ventilatory limitation in spirometry has been described in one-third or less of the subjects upon hospital discharge,^2 , 3^with lower incidence in medium-term follow-up.^4 , 5^Conversely, diffusion abnormalities were described at a higher rate, mainly in individuals with more severe illness.^3 , 4 , 6^Many myocardial injury cases have also been described in more severely affected subjects.^7 , 8^Moreover, after acute phase recovery, there is an increasing report of persistent symptoms, and this clinical condition has been referred to as “post-acute COVID-19 syndrome” or “long COVID”.^1 , 9^Nevertheless, limited information is available concerning the underlying causes of these persistent symptoms.^1^

Cardiopulmonary exercise test (CPET) is a well-established diagnostic tool to assess symptom etiology and underlying mechanisms limiting exercise in cardiovascular and pulmonary diseases.^10^An initial study with CPET assessment in post-COVID-19 subjects found a reduction in peak oxygen uptake (VO_2_) achieving 66.2 ± 10.5% of the predicted values at 1-month post-discharge, despite spirometry being within the normal range. A reduction in oxygen pulse was also observed in 70% of the subjects, while 80% presented normal carbon dioxide ventilatory equivalent (VE/VCO_2_) values, strengthening extrapulmonary factors, such as hospitalization “bed rest effect”, as the possible etiology of exercise limitations.^11^Similarly, a study with eighteen subjects reported a 30% reduction in peak VO_2_but an increased VE/VCO_2_, due possibly to increased chemosensitivity and reduced oxygen content and extraction as the main contributors to reduced exercise capacity.^12^Raman et al.^5^evaluated fifty-one subjects after hospital discharge and found a reduction in peak VO_2_and the oxygen efficiency slope (OUES), as well as increased VE/VCO_2_upon the termination of CPET due mostly to generalized muscle aches and fatigue rather than breathlessness, suggesting deconditioning and peripheral skeletal muscle impairments as the probable cause of exercise limitation. Clavario et al.^13^also reported functional limitations, mainly due to muscular impairment, in one-third of pos-COVID-19 subjects at three months after hospital discharge.

However, all previous studies examining CPET-related outcomes in post-COVID-19^5 , 11 , 12^were performed after hospital discharge in patients with a greater severity of illness. Thus, the impact of COVID-19 on CPET in mild to moderately severe illness is limited. For this reason, a better understanding of exercise limitation in post-COVID-19 outpatients with a broader clinical profile is needed.

Thus, our primary objective was to characterize the CPET abnormalities in severity subgroups of post-COVID-19 subjects with a broad clinical spectrum and compare them to a matched control group. The secondary objective was to compare post-COVID-19 CPET measures to results obtained before infection to better understand the effects of COVID-19 on exercise tolerance.

## Methods

### Participants

Our study conducted a retrospective cohort study of outpatients referred for CPET assessment at an experienced laboratory in the Brazilian Midwest region from June 2020 to August 2021. The inclusion criteria were a clinical history of symptomatic COVID-19, confirmed by real-time reverse transcription–polymerase chain reaction, absence of previous cardiovascular or pulmonary disease, and referral for CPET due to persistent symptoms or to exclude cardiopulmonary dysfunction in the post-acute illness. A control group was selected by propensity score matching among the tests performed on healthy individuals, without previous cardiopulmonary diseases and without COVID-19 during the same time period to assure similar restrictions due to mitigation strategies. In addition, for pairwise comparison (before and after COVID-19), previous CPETs were searched on the laboratory database from January 2011 to February 2020, with only the most recent CPET considered if more than one exam was found. All exams were performed by the same cardiologist, certified by the Brazilian Society of Cardiology. Institutional review board approval was obtained from the Ethics Review Board (CAAE: 35706720.4.0000.8093) on September 16, 2021. The informed consent term was waived as data were collected retrospectively.

### Clinical assessment

Medical evaluations were performed before the CPET and clinical information regarding comorbidities (cardiovascular risk factors and previous cardiopulmonary diseases), medications, and demographic characteristics were obtained. COVID-19 related clinical information was also collected, i.e., symptom onset date, manifestations during the acute viral disease, computed tomography (CT) images, and treatment facility used for this study.

### COVID-19 illness severity criteria

The severity of COVID-19 was classified according to clinical and imaging characteristics: *mild* - individuals with any of the various signs and symptoms of COVID-19, but who do not have shortness of breath, dyspnea, abnormal chest imaging, or reduced oxygen saturation; *moderate* - individuals who show evidence of lower respiratory disease during clinical assessment or imaging and who have oxygen saturation above 94% on room air; *severe* - individuals who have oxygen saturation below 94% on room air, respiratory frequency > 30 breaths/min, or lung infiltrates > 50%; *critical* - individuals who have respiratory failure, septic shock, or multiple organ dysfunction. In addition to pulmonary disease, patients with critical illness may have also experienced cardiac, hepatic, renal, central nervous system, or thrombotic disease.^1^

### Cardiopulmonary exercise testing

CPET was performed on a treadmill (Centurium 200) with breath-by-breath gas analysis (Metalyzer 3B, Cortex). Symptom-limited maximal exercise testing with an individualized ramp protocol was used to yield a fatigue-limited exercise duration of 8 to 12 min.^10 , 14^Precautions to mitigate viral transmission were adopted following national recommendations.^15^

The following CPET variables were obtained:

- pre-effort spirometry: forced expiratory volume in one second (FEV1) and forced vital capacity (FVC).
- clinical signs and symptoms, electrocardiographic monitoring, and pulse oximetry.
- peak heart rate (HR, bpm) and HR at ventilatory thresholds (VT) 1 and 2.
- peak oxygen uptake (peak VO_2_, L/min and mL/kg/min).
- percent-predicted peak VO_2_(VO_2_%).
- VO_2_(mL/kg/min) at VT1 and VT2.
- peak oxygen pulse (mL/beat).
- peak respiratory exchange ratio.
- peak loads achieved (speed, km/h, and inclination, %).
- peak minute ventilation (VE, L/min and percent-predicted) and ratio of VE to maximal voluntary ventilation (VE/VVM).
- peak VE/VO_2_.
- carbon dioxide ventilatory equivalent (VE/VCO_2_) slope.
- oxygen uptake efficiency slope (OUES).

Peak VO_2_and minute ventilation were expressed as the highest 30-second averaged sample obtained from the effort final minute. The highest achieved values at the peak effort were considered for other peak variables. VT1 and VT2 were determined by an experienced physician, and VE/VCO_2_slope was calculated until VT2. Predicted HR was calculated by the formula 220 – age (yr)^16^and predicted peak VO_2_according to the Brazilian Midwest reference values.^17 , 18^Cardiorespiratory fitness (CRF) was classified according to the percentile distribution of peak VO_2_in Brazilian Midwest reference values,^19^which identified reduced CRF if measured values were below the 5^th^percentile. Percent-predicted peak VE was calculated according to a prediction equation.^20^MVV was calculated as measured or predicted by FEV1 x 40. Brazilian reference values were used for spirometry.^21^

The etiology of exercise limitation was identified by clinical interpretation of the CPET variables according to the Wasserman 9-panel plot analyses and the European Respiratory Society criteria as well as patient signs and symptoms.^10 , 22 - 24^

In the sample with CPET prior to viral infection available, a longer interval between the before and after assessments is possible. Thus, to verify the aging influences in the post-COVID peak VO_2_, calculations of the differences in the age-related predicted values in both CPETs were performed according to the Brazilian reference values.^18^

### Statistical analysis

Categorical variables were described using absolute and relative frequencies and continuous variables were not normally distributed, being described through median and interquartile range. The normality of the data was examined by the Shapiro-Wilk test. Wilcoxon signed-rank tests were used to compare pairwise continuous variables (within-group). Mann-Whitney or Kruskal-Wallis with Muller Dunn post hoc tests compared subgroup variables as appropriate. Median difference and 95% confidence intervals (CI) were calculated by Hodges-Lehmann estimates. Chi-square tests examined categorical variables. Propensity score matching was used to obtain the healthy control subgroup paired to the COVID-19 subjects. Propensity scores were estimated according to the predictors: sex, age, weight, height, and body mass index (BMI). A one-to-one matched pair selection, using nearest-neighbor matching, was performed based on the estimated propensity scores of each subject, and the match tolerance (caliper) was set as 0.10. Multiple logistic regression was performed to analyze the capacity of several independent variables, age, sex, COVID-19 severity, and presence of obesity, to predict a reduced CRF. Relative risks (RR) and 95% CI were calculated. A two-sided p-value < 0.05 was considered significant for all analyses. Statistical analysis was performed using IBM-SPSS 28.0.

## Results

### Study sample

During the study period, a total of 867 CPET were performed ( Figure 1 ), and a clinical history of symptomatic COVID-19 was identified in 167 subjects. After excluding subjects with previous cardiovascular or pulmonary diseases, a sample of 144 subjects was included in the study [age 43 (36, 53) yr, 57% male] ( Table 1 ). Illness severities were classified as mild in 87 (60%), moderate in 30 (21%), severe in 25 (17%), and critical in 2 (1%). The two critical illness subjects were combined with the severe subgroup for group analysis. The control subgroup (n = 144) was selected by propensity score matching from 322 healthy adults without COVID-19 ( Figure 1 ).

**Figure 1:** – Flowchart diagram of patients

**Table 1:** – Demographic characteristics, comorbidities, and prior medications in control subjects and in COVID-19 subjects by illness severity

Characteristics	Control subjects	COVID-19 subjects						p Value*
		Overall			Mild	Moderate	Severe	
Subjects	144 (100)		144 (100)		87 (60)	30 (21)	27 (19)	
**Demographics**								
Male	83 (58)		82 (57)		48 (55)	16 (53)	18 (67)	.722
Female	61 (42)		62 (43)		39 (45)	14 (47)	9 (33)	
Age, yr	43.0 (38.0, 51.8)		43.0 (36.0, 53.0)		40.0 (33.0, 49.0)	46.5 (40.8, 53.0)	54.0 (44.0, 61.0)	< .001
Weight, kg	76.3 (65.7, 86.7)		77.2 (67.1, 85.7)		73.1 (65.0, 83.0)	84.4 (69.1, 95.3)	81.1 (73.1, 88.1)	.026
Height, m	1.71 (1.63, 1.78)		1.70 (1.63, 1.78)		1.70 (1.61, 1.76)	1.70 (1.63, 1.78)	1.74 (1.66, 1.78)	.68
BMI, kg/m^2^	25.9 (23.5, 29.2)		26.0 (23.7, 28.6)		25.0 (23.1, 27.9)	27.7 (25.4, 30.3)	28.3 (26.1, 29.4)	< .001
Hypertension	0 (0)		21 (15)		10 (12)	4 (13)	7 (26)	< .001
Diabetes mellitus	0 (0)		2 (1)		0 (0)	0 (0)	2 (7)	< .001
**COVID-19 acute phase information**								
SpO_2_< 94% on room air			22 (15)		0 (0)	0 (0)	22 (82)	< .001
Lung infiltrates on CT								
≥ 50%			18 (13)		0 (0)	0 (0)	18 (67)	< .001
25-49%			21 (15)		0 (0)	15 (50.0)	6 (22)	
≤ 24%			17 (12)		0 (0)	15 (50.0)	2 (7)	
Normal			25 (17)		25 (29)	0 (0)	0 (0)	
Not performed			63 (44)		62 (71)	0 (0)	1 (4)	
Hospitalization			22 (15)		0 (0)	1 (3)	21 (78)	< .001
Mechanical ventilation			2 (1)		0 (0)	0 (0)	2 (7)	.012
Persistent symptoms related to COVID-19			60 (42)		23 (26)	14 (47)	23 (85)	< .001

Values are expressed as median and interquartile range or absolute and relative frequencies, as appropriate. Statistical analysis: Comparison between control and overall COVID-19 subjects: Mann-Whitney Test: all demographic variables were not different (effective pairing). * Comparisons between illness severity subgroups and control subjects: Kruskal-Wallis test for continuous variables or Chi-square for categorical variables. BMI: body mass index; CT: computed tomography; SpO_ 2_: oxygen saturation.

Most of the patients were previously healthy, with a low frequency of hypertension ( Table 1 ). During the COVID-19 acute phase, the hospitalization rate was low, with only 2 patients requiring mechanical ventilation, although some patients had oxygen desaturation at rest and abnormalities on pulmonary CT. Residual symptoms related to post-COVID-19 syndrome (such as fatigue, shortness of breath, dyspnea, and reduced exercise tolerance) were identified in 60 subjects (42%), with increased frequency according to illness severity ( Table 1 ).

Post-COVID-19 and control subjects were not different in age, sex, and anthropometric data, indicating an effective matching strategy. However, in comparing illness severities and controls, moderate and severe illness subgroups were older and had greater weight and BMI than those with mild illness and controls ( Table 1 ). Among post-COVID-19 subgroups, a lower prevalence of hypertension and medication use were observed in subjects with milder illness than higher severity illness during the acute phase.

### Cardiopulmonary Exercise Test

CPET was performed on average 11.5 (7.0, 21.2) weeks after the acute disease, with the majority of CPET limited by peripheral muscle fatigue (92%), with minimal limitation due to the pulmonary and cardiovascular systems (6% and 2%, respectively) ( Table 2 ). Although peripheral muscular fatigue was the predominant etiology of exercise limitation, the magnitude of CRF reduction varied among COVID-19 illness severities and was lower in moderate and severe subgroups than in subjects with mild illness and controls. Significantly lower VO_2_% was observed in the severe subgroup compared to both mild and control subgroups ( Figure 2 ).

**Table 2:** – Cardiopulmonary exercise test variables in COVID-19 subjects according to illness severity and control subjects

Characteristics	Control subjects (n=144)		COVID-19 subjects				p Value*
			Overall (n=144)	Mild (n=87)	Moderate (n=30)	Severe (n=27)	
**CPET variables**							
Time after COVID-19 acute disease, wk	--	11.5 (7.0, 21.2)		11.4 (6.7, 22.3)	10.5 (6.5, 15.6)	13.6 (7.9, 23.3)	.288
Exercise duration, min	10.0 (8.9, 1.3)	10.4 (9.0, 1.3)		10.2 (9.0, 11.4)	10.3 (8.8, 11.3)	10.7 (9.4, 11.7)	.551
Peak HR, bpm	171 (162, 180)	172 (160, 180)		178 (169, 185)	170 (155, 179)	156 (147, 169)	< .001 † .001; ‡ < .001; ‖ .018
Peak HR, % predicted	98.3 (94.0, 101.7)	97.2 (93.2, 101.7		98.3 (94.5, 102.3)	96.7 (93.1, 101.3)	94.0 (87.0, 100.0)	0.118
Peak speed, km/h	9.5 (7.6, 11.9)	9.2 (7.3, 11.5)		10.6 (8.5, 12.3)	9.3 (6.9, 11.7)	7.1 (6.6, 7.9)	< .001 † ‡ < .001; § .024
Peak inclination, %	3.5 (3.0, 4.0)	3.5 (3.0, 4.0)		3.5 (3.0, 4.0)	3.5 (3.0, 4.0)	4.5 (3.5, 6.0)	< .001 † .001; ‡ < .001; § .005
Peak RER	1.17 (1.11, 1.22)	1.13 (1.08, 1.21)		1.14 (1.08, 1.22)	1.11 (1.05, 1.18)	1.13 (1.10, 1.22)	.017 ¶ .014
Peak VO_2_, L/min	2.49 (1.76, 3.11)	2.40 (1.85, 2.98)		2.60 (1.95, 3.16)	2.46 (1.67, 3.03)	2.06 (1.48, 2.57)	.026 ‡ .016
Peak VO_2,_mL/kg/min	30.9 (25.3, 38.4)	31.1 (23.6, 37.5)		35.7 (28.1, 40.1)	29.9 (22.7, 35.3)	22.6 (20.4, 29.1)	< .001 † ‡ < .001
Peak VO_2_, % predicted	91.6 (78.2, 111.8)	92.4 (76.7, 107.4)		98.5 (86.5, 111.8)	87.1 (74.9, 103.6)	72.2 (64.1, 89.4)	< .001 † .001; ‡ < .001
Peak oxygen pulse, mL/beat	14.4 (10.6, 18.4)	14.0 (11.1, 17.4)		14.5 (11.3, 18.2)	14.5 (10.5, 17.5)	13.5 (11.1, 15.1)	.558
OUES	2,544 (1,900, 3,246)	2,570 (1,968, 3,236)		2,707 (2,035, 3,389)	2,742 (1,767, 3,248)	2,240 (1,729, 2,767)	.134
Peak VE, L/min	100.1 (70.9, 128.1)	94.8 (75.6, 118.8)		98.5 (77.9, 125.2)	95.5 (77.1, 112.5)	77.0 (64.9, 100.9)	.067
Peak VE, % predicted	106.2 (92.4, 118.7)	104.9 (93.6, 115.5)		104.9 (96.0, 116.6)	108.8 (91.8, 115.6)	94.3 (75.1, 111.7)	.103
Peak VE/MVV, %	71.3 (59.7, 83.7)	72.0 (63.1, 81.1)		71.9 (63.3, 80.9)	74.2 (65.0, 81.5)	71.1 (56.6, 84.1)	.888
Peak VE/VO_2_	40.2 (37.0, 43.3)	40.2 (36.4, 44.0)		39.8 (36.1, 44.0)	40.4 (36.9, 42.6)	42.1 (37.4, 46.2)	.503
VE/VCO_2_slope	32.5 (29.7, 35.1)	33.8 (30.6, 36.4)		33.6 (30.0, 35.5)	34.0 (31.4, 37.4)	34.0 (30.6, 39.0)	.089
VO_2_at VT1, mL/kg/min	15.8 (13.4, 23.2)	15.6 (12.9, 23.1)		17.8 (13.5, 24.5)	14.3 (11.7, 20.3)	13.1 (12.0, 14.5)	< .001 † .002; ‡ < .001
Exercise HR at VT1, bpm	120 (112, 129)	120 (112, 130)		124 (114, 137)	116 (109, 126)	112 (106, 120)	< .001 ‡ < .001
VO_2_at VT2, mL/kg/min	27.5 (21.0, 34.2)	28.5 (22.3, 35.0)		32.8 (25.7, 36.4)	27.4 (20.7, 33.4)	21.0 (17.5, 26.1)	< .001 † .001; ‡ < .001; ‖ .036
Exercise HR at VT2, bpm	158 (146, 167)	162 (151, 172)		167 (158, 174)	158 (152, 167)	142 (135, 154)	< .001 † .002; ‡ ‖ < .001; § .011
Exercise SpO_2_reduction	0 (0)	7 (4.9)		0 (0)	0 (0)	7 (25.9)	< .001
**CPET termination reason**							
Muscle fatigue	144 (100)	132 (92)		85 (98)	28 (93)	19 (70)	< .001
Cardiovascular limitation	0 (0)	3 (2)		2 (2)	0 (0)	1 (4)	
Pulmonary limitation	0 (0)	9 (6)		0 (0)	2 (7)	7 (26)	
Total	144 (100)	144 (100)		87 (100)	30 (100)	27 (100)	

Values are expressed as median and interquartile range or absolute and relative frequencies. * Comparisons between illness severity subgroups and control subjects: Kruskal-Wallis test with Muller Dunn post hoc test for continuous variables or Chi-square for categorical variables. Post-test p values reported when Kruskal-Wallis p value < .05: † Severe versus Control; ‡ Severe versus Mild; § Severe versus Moderate; ‖Mild versus Control; ¶ Moderate versus Control; # Moderate versus Mild. CPET: cardiopulmonary exercise test; HR: heart rate; MVV: maximal voluntary ventilation; OUES: Oxygen uptake efficiency slope; RER: respiratory exchange ratio; SpO_2_: oxygen saturation; VCO_2_: carbon dioxide production; VE: minute ventilation; VO_2_:oxygen uptake; VT: ventilatory threshold.

**Figure 2:** – Cardiopulmonary exercise test (CPET) variables in subjects with COVID-19 according to illness severity and control subjects. Subjects with CPET after COVID-19 (n = 144) and controls (n = 144). Values are expressed as median, interquartile range, and limits. HR: heart rate; OUES: oxygen uptake efficiency slope; RER: respiratory exchange ratio; VCO2: carbon dioxide production; VE: minute ventilation; VO2: oxygen uptake; VT: ventilatory threshold.

Moderate severity subjects exhibited a median of peak VO_2_5.8 mL/kg/min lower than mild illness subgroup, and 1.0 mL/kg/min lower than controls, although the differences were not significant. Severe illness subjects presented greater limitations and significantly lower median peak VO_2_compared to the mild illness subgroup and controls (13.1 and 8.3 mL/kg/min, respectively) ( Table 2 and Figure 2 ). As a result, CRF classification was significantly different among the severity subgroups ( Figure 3A ), with reduced CRF in 56% of the severe illness subjects and lower frequencies in the moderate and mild subgroups (23% and 10%, respectively).

**Figure 3:** – Cardiorespiratory fitness (CRF) classification and COVID-19 severity. A) Data for subjects with cardiopulmonary exercise test (CPET) after COVID-19 (n = 144) and controls (n = 144); B) Data for a subgroup with CPET before and after the viral disease (n = 42). Illness severity values presented as percentage of distribution (%) and CRF as classification percentile, according to peak oxygen uptake.

According to multiple logistic regression, age and sex were not able to predict a reduced CRF, while both COVID-19 severity and obesity were (r2 = 0.46). Subjects with severe COVID-19 had a higher risk of presenting a reduced CRF [RR: 8.6 (95% CI: 2.9 to 25.7)] compared to the controls. By contrast, mild or moderate illness severities were not associated with a lower CRF. Similarly, obese and overweight classification were identified as a robust predictor of a reduced CRF [RR: 37.4 (95% CI: 11.7 to 119.9) and [RR: 3.4 (95% CI 1.07 to 11.1), compared to healthy weight subjects.

Regarding the ventilatory thresholds, significantly lower VO_2_at VT1 and VT2 were also found in more severely ill subjects ( Table 2 and Figure 2 ). Likewise, the HR at peak and at VTs were similar, with significantly lower values in the severe illness subgroup. Peak minute ventilation, OUES, and peak oxygen pulse revealed median values that were lower in the severe illness subjects, but the differences among the other subgroups and controls were not statistically significant. All other CPET variables were also not significantly different among COVID-19 illness severity subgroups ( Table 2 and Figure 2 ).

Resting spirometry performed before CPET found normal values in several post-COVID-19 subjects with mild abnormalities in only 13% of the subjects based on FVC values and in 14% based on FEV1 values. Although not statistically significant, severe illness subjects presented a higher proportion of mild abnormalities based on FVC and FEV1 values (29% and 18%, respectively). However, the percent-predicted values were significantly lower in severe illness subjects than in controls (FVC: 85.7 versus 99%, p = 0.022 and FEV1: 85.5 versus 94.3%, p = 0.007).

### Subgroup of patients with previous CPET assessment

Forty-two of the 144 subjects (29%) had a previous CPET available for comparison ( Figure 1 ). This subgroup of subjects had COVID-19 severity classified as mild in 27 subjects (64%), moderate in 9 (21%), and severe in 6 (14%). For severity subgroup analysis, the moderate and severe subjects were combined. Characteristics of these 42 subjects were similar to the full study sample ( Table 3 ). During the COVID-19 acute phase, the hospitalization rate was also low, and mechanical ventilation was not required in any subjects.

**Table 3:** – Demography, comorbidities, prior medications, and COVID-19 clinical information in a subgroup of subjects with previous evaluation

Characteristics	COVID-19 subjects			p Value *
	Overall	Mild	Moderate/severe	
Subjects	42 (100)	27 (64)	15 (36)	
**Demographics**				
Male	27 (64)	17 (63)	10 (67)	.810
Female	15 (36)	10 (37)	5 (33)	
Age, years	46.5 (40.0, 54.0)	43.0 (36.0, 51.0)	52.0 (44.0, 55.0)	.030
Weight, kg	81.4 (70.4, 85.2)	82.0 (69.2, 85.2)	81.3 (73.3, 87.0)	.906
Height, cm	173.5 (165.0, 178.0)	175.0 (168.0, 180.0)	173.0 (164.0, 178.0)	.264
BMI, kg/m^2^	25.8 (23.4, 27.5)	25.2 (23.0, 27.3)	26.4 (24.0, 27.8)	.259
Hypertension	8 (19)	6 (22)	2 (13)	.482
Diabetes mellitus	0 (0)	0 (0)	0 (0)	--
**COVID-19 acute phase information**				
SpO2<94% on room air	4 (10)	0 (0)	4 (27)	.005
Hospitalization	4 (10)	0 (0)	4 (27)	.005
Persistent symptoms related to COVID-19	14 (33)	6 (22)	8 (53)	.040

Values are expressed as median and interquartile range or absolute and relative frequencies. Statistical analysis: * Comparisons between illness severity subgroups: Mann-Whitney test for continuous variables or Chi-square for categorical variables. BMI: body mass index; SpO_2_:oxygen saturation.

The CPET before COVID-19 infection was performed with a median interval of 25.0 (16.8, 40.0) and 39.4 (19.9, 67.5) months in the mild and moderate/severe illness subgroups, respectively, and this difference was not significant.

In the mild illness subgroup of 27 subjects, the CPET before and after the COVID-19 infection did not reveal significant changes in the majority of measures except for a median reduction in peak treadmill speed of 0.4 km/h [95% CI: - 0.6 to - 0.2], a small reduction in exercise duration of 0.5 minutes, and a small increase in percent-predicted peak HR ( Table 4 and Figure 4 ).

**Table 4:** – Cardiopulmonary exercise test variables in subgroup with previous exam and division according to COVID-19 severity

	COVID-19 severity					
	Mild (n = 27)			Moderate or severe (n = 15)		
**CPET variables**	**Before**	**After**	p Value * Difference †	**Before**	**After**	p Value * Difference †
Exercise duration, min	10.6 (9.9, 11.3)	10.4 (9.0, 11.1)	-0.5 (-1.0 to -0.05) p = .037	11.3 (9.9, 12.4)	10.4 (9.0, 11.3)	-1.0 (-1.8 to -0.2) p = .022
Peak HR, bpm	173 (163, 181)	173 (160, 185)	0.5 (-2.5 to 3.0) p = .829	173 (168, 178)	169 (156, 176)	-5 (-10 to -0.5) p = .029
Peak HR, % predicted	96.3 (93.5, 99.4)	97.3 (94.4, 102.3)	1.7 (0.2 to 3.1) p =.027	97.8 (96.1, 102.9)	98.1 (93.7, 101.7)	-1.1 (-4.1 to 1.6) p = .49
Peak speed, km/h	11.9 (11.0, 14.1)	11.7 (10.6, 14.2)	-0.4 (-0.6 to -0.2) p = .005	11.2 (8.2, 13.2)	9.5 (6.8, 12.5)	-1.0 (-1.5 to -0.6) p = .001
Peak inclination, %	3.5 (3.0, 3.5)	3.5 (3.0, 3.5)	-0.3 (-0.5 to 0) p =.048	4.0 (3.5, 4.0)	3.5 (3.0, 4.0)	-0.3 (-0.8 to 0.3) p = .259
Peak RER	1.10 (1.06, 1.21)	1.14 (1.08, 1.22)	0.03 (-0.02 to 0.07) p = .218	1.13 (1.11, 1.17)	1.17 (1.10, 1.24)	0.04 (-0.02 to 0.09) p = .196
Peak VO_2_, L/min	2.93 (2.00, 3.72)	3.08 (2.09, 3.48)	-0.05 (-0.17 to 0.05) p = .249	2.29 (2.04, 3.28)	2.12 (1.84, 2.96)	-0.16 (-0.38 to -0.03) p = .023
Peak VO_2_, mL/kg/min	40.8 (33.7, 44.7)	38.0 (33.4, 41.0)	- 0.8 (-2.2 to 0.3) p = .175	34.1 (26.5, 40.5)	27.8 (22.3, 36.0)	-4.1 (-9.8 to -2.3) p < .001
Peak VO_2,_% predicted	110.4 (99.8, 122.1)	106.3 (98.2, 124.8)	-0.4 (-3.7 to 2.8) p = .866	108.1 (83.9, 113.6)	90.1 (69.6, 103.9)	-7.4 (-20.5 to -0.7) p =.041
Peak oxygen pulse, mL/beat	17.7 (11.8, 21.6)	17.6 (13.0, 19.8)	-0.3 (-1.1 to 0.4) p = .414	14.4 (11.8, 19.1)	13.6 (11.1, 16.6)	-0.9 (-2.3 to 0.5) p = .256
OUES	3,075 (2,176, 3,706)	2,977 (2,283, 3,554)	-115 (-262 to 56) p = .121	2,481 (2,209, 3,215)	2,260 (2,086, 3,047)	-140 (-517 to 37) p = .078
Peak VE, L/min	121.3 (89.1, 139.4	119.2 (90.8, 139.3)	-0.7 (-5.4 to 2.8) p = .639	98.4 (80.8, 126.3)	95.3 (78.5, 127.4)	-5.3 (-11.1 to 2.2) p = .147
Peak VE, % predicted	112.8 (101.9, 120.8)	114.2 (105.1, 125.4)	1.6 (-2.1 to 5.2) p = .374	114.2 (95.1, 122.5)	111.1 (95.9, 116.2)	- 2.8 (-10.1 to 6.3) p = .570
Peak VE/MVV, %	74.9 (67.4, 81.7)	77.9 (71.9, 85.7)	2.3 (-0.7 to 10.2) p = .097	72.80 (62.8, 79.2)	78.1 (71.2, 89.0)	7.7 (-2.0 to 18.3) p = .100
Peak VE/VO_2_	39.6 (36.9, 42.4)	40.0 (36.5, 42.9)	0.4 (-0.8 to 1.5) p = .360	39.1 (37.6, 45.3)	42.4 (40.2, 45.0)	1.7 (-0.3 to 3.2) p = .167
VE/VCO_2_slope	33.9 (31.6, 36.0)	34.2 (30.6, 35.5)	-0.4 (-1.9 to 1.4) p = .631	31.4 (29.6, 36.8)	33.1 (31.5, 34.9)	-0.05 (-2.8 to 3.4) p = .977
VO_2_at VT1, mL/kg/min	25.2 (18.8, 28.1)	23.9 (18.5, 26.3)	-1.6 (-3.4 to 0.7) p = .133	19.8 (13.7, 25.6)	14.9 (12.8, 23.9)	-2.1 (-4.1 to -0.7) p = .011
HR at VT1, bpm	137 (119, 143)	129 (116, 143)	-2 (-7 to 4) p = .400	124 (112, 130)	117 (112, 126)	-6 (-13 to 2) p = .094
VO_2_at VT2, mL/kg/min	38.3 (29.6, 42.8)	35.1 (32.7, 37.5)	-1.6 (-3.6 to 0) p = .05	30.7 (23.8, 37.4)	27.4 (21.8, 33.1)	-3.2 (-6.8 to -1.7) p = .003
Exercise HR at VT2, bpm	164 (158, 174)	166 (151, 174)	-1 (-4 to 3) p =.637	159 (156, 165)	156 (146, 165)	-5 (-10 to 1) p = .069

Values are expressed as the median and interquartile range. Statistics: * Wilcoxon signed-rank test was used to compare before and after variables within each COVID-19 severity subgroup. † Median differences (after minus before) and 95% CI estimates calculated by Hodges-Lehman method. CPET: cardiopulmonary exercise test; HR: heart rate; MVV: maximal voluntary ventilation; RER: respiratory exchange ratio; VO_2_:oxygen uptake; VCO_2_: carbon dioxide production; VE: minute ventilation; VT: ventilatory threshold; OUES: Oxygen uptake efficiency slope.

**Figure 4:** – Cardiopulmonary exercise testing (CPET) variable differences according to COVID-19 severity. Patient subgroup (n = 42) with CPET before and after COVID-19. Values are median difference and 95% CI. HR: heart rate; OUES: oxygen uptake efficiency slope; RER: respiratory exchange ratio; VCO2: carbon dioxide production; VE: minute ventilation; VO2: oxygen uptake; VT: ventilatory threshold. Some variables were adjusted for graphic scaling. Peak VO2(L/min), peak RER, and peak speed (km/h), multiplying by 10; OUES, multiplying by 10-2.

In the moderate/severe subgroup of 15 subjects, a significant median reduction in peak speed was observed, but with a greater magnitude [1.0 km/h (95% CI: - 1.5 to - 0.6). Furthermore, significant reductions in VO_2_at peak and at the VTs, were observed. The median reduction in peak VO_2_of 4.1 mL/kg/min (95% CI: - 9.8 to - 2.3) reflected a 7.4% (95% CI: -20.5 to - 0.7) reduction in peak VO_2_% (Table 4 and Figure 4 ). Similarly, a reduced level of CRF was previously identified in 13% of the subjects which increased to 33% after COVID-19 ( Figure 3B ). Considering the age-related reduction in peak VO_2_due to the interval between the CPET assessments, the predicted values median difference was -0.8 (-0.7 to -1.4) mL/kg/min, which accounted for only about 20% of the observed median peak VO_2_reduction.

Peak HR was also significantly lower after COVID-19, with a median difference of -5 beats per minute (95% CI: - 10 to - 0.5). Despite the significant changes observed in peak VO_2_and HR, there were no significant differences in ventilatory equivalents, OUES, peak oxygen pulse, peak minute ventilation, and other CPET variables before and after COVID-19 in the moderate/severe subgroup.

## Discussion

To the best of our knowledge, this is one of the largest studies to examine CPET results in post-COVID-19 subjects, presenting with a broad clinical spectrum, and has compared CPET results to a control group selected by propensity score matching. Furthermore, the comparison of CPET performed before COVID-19 in almost 30% of the study cohort is noteworthy and strengthens the relevance, novelty, and importance of the study findings.

Our results indicate that patients who experienced severe COVID-19 illness have a significant reduction in CRF three months after symptom onset, confirmed by a lower median peak VO_2_than subjects with mild illness and control subjects. Pairwise comparison of subjects with a previous CPET prior to COVID-19 supports the findings in the entire cohort. Additionally, the 9-pannel plot and signs and symptoms observed during CPET reveal that peripheral muscle fatigue was the most prevalent cause of exercise limitation in subjects post-COVID-19. These findings are similar to previous studies, including hospitalized subjects.^5 , 11 - 13^

Recent CPET studies in post-COVID-19 subjects found a similar reduction in peak VO_2_of 30% to 34% of the predicted values.^5 , 11 , 12^Extrapulmonary factors, such as hospitalization, “bed rest”,^11^anemia,^12^and muscle weakness,^5^were presented as possible underlying mechanisms for reduced CRF. Our results support such extrapulmonary factors, since subjects with moderate and severe illness presented peak VO_2_% values that were 11.4% and 26.3% lower than the mild illness subgroup ( Table 2 ), highlighting the role that peripheral muscle fatigue had on limiting exercise in our cohort of mainly non-hospitalized subjects post-COVID-19. Furthermore, in the 42 patients with CPET before COVID-19, significant reductions in peak VO_2_and peak VO_2_% were observed only in subjects with higher severity illness, which could not be explained by the age difference between CPET assessments. In view of the above, our findings and those of others have identified the major detrimental effects severe COVID-19 has on exercise tolerance and skeletal muscle performance.^5 , 12 , 13 , 25^

Similar to previous studies with hospitalized patients, reduced CRF due to peripheral skeletal muscle impairment from abnormal peripheral oxygen extraction due to muscle catabolism seems to be a more likely consequence of COVID-19 rather than a “bed rest” effect.^12^This mechanism is strengthened by the marked reductions observed in VO_2_at both VT1 and VT2, comparing illness severities and controls ( Table 2 and Figure 2 ), as well as the pairwise comparison before and after COVID-19 in mostly non-hospitalized subjects ( Table 4 and Figure 4 ). These observations indicate early activation of anaerobic metabolism and lower buffering capacity during exercise,^10^which may be one of the key mechanisms responsible for persistent fatigue symptoms in patient’s post-COVID-19. Similarly, Singh et al.^25^using invasive CPET assessment, reported a marked reduction in peak VO_2_associated with impaired oxygen extraction, despite a preserved cardiac index, reinforcing a peripheral rather than central cardiac limit during exercise.

Moreover, we observed a significantly lower peak HR in subjects with severe illness compared to controls and the subgroup of individuals with mild illness ( Figure 2 ), as well as a lower peak HR during the post-COVID-19 CPET when compared to CPET before COVID-19 in the moderate/severe illness subgroup ( Figure 4 ). This finding is supported by a previous study in COVID-19 subjects^12^that reported a similar reduction in peak HR. We also found that HR at VT1 and VT2 were significantly lower in the severe illness subgroup when compared to the mild illness and control subgroups, but such a reduction was not observed in the pairwise comparison. The combination of lower peak HR and peak VO_2_values and early VT may be attributed to a metabolic myopathy limiting exercise^22^which corroborates muscle fatigue as the main cause for exercise limitation in our study. It is important to highlight that these differences in peak values were not associated with lower relative effort intensity in that the peak respiratory exchange ratio was not different among the subjects within different illness severities and controls, nor during pairwise before-after COVID-19 comparisons.

Despite the reported differences in VO_2_and HR at the peak and VT1 and VT2, no other CPET measure was significantly different among the illness severity subgroups and controls ( Table 2 and Figure 2 ) nor during pairwise before-after COVID-19 comparisons. Despite the pulmonary involvement in more severely affected subjects during acute COVID-19 infection and a 42% rate of residual symptoms, we found no differences in ventilatory efficiency, quantified by the VE/VCO_2_slope, neither in the matched-pair comparisons with the control group ( Table 2 and Figure 2 ) nor in the pairwise analysis with pre-COVID-19 data ( Table 4 and Figure 4 ) which are similar to the results reported by Gao et al.^11^.

However, several other studies^5 , 12 , 25^have reported higher VE/VCO_2_slope values in COVID-19 subjects compared to controls, which, according to Baratto et al.^12^were attributed to increased chemosensitivity stimulating higher ventilation. Also different from our results is that lower values for the OUES^5^and oxygen pulse^11 , 12^have been reported in post-COVID-19 subjects. However, the subjects in these studies were assessed at or soon after hospitalization, with a higher possibility of myocardial injury and dysfunction,^8 , 26^which could have influenced the CPET results reported in these studies. In our study, post-COVID-19 patients with a broad clinical spectrum were evaluated, and only 15% of the subjects had been previously hospitalized during the acute viral phase. Characteristics of our participant sample may have produced the different results in these CPET variables.

According to multiple logistic regression, we identified that severe COVID-19 was associated with a nearly 9-fold higher risk of presenting a reduced CRF, highlighting the impact of disease severity on the exercise limitation. While both age and sex were not predictors, obesity was a robust predictor with a 37-fold increased risk. The impact of weight and illness severity on the exercise limitation was also demonstrated by Braga et al.^27^; however, in this study, age and sex were predictors of reduced CRF, which is opposite to our findings.

Finally, it is essential to highlight that the main findings of the present study indicate that the primary reason for exercise limitation, and probably the reason for the persistent symptoms, was peripheral muscle limitation in the majority of the subjects (92%). Pulmonary and cardiovascular limitations were identified in only 12 subjects (8%) who were most severely affected. In the severe subgroup, the main limitation was also peripheral muscle fatigue (70%), of whom 85% reported persistent symptoms. However, 26% of the patients in this subgroup presented significant reductions in pulse oximetry during exercise despite the majority having normal spirometry. This finding embodies the importance of using CPET in subjects with persistent symptoms, especially in those more severely affected by COVID-19, since normal or near-normal resting spirometry may not be enough to exclude pulmonary dysfunction in post-COVID-19 patients.

### Study limitations

This study has several limitations that should be addressed. First, it is a unicentric retrospective study performed in one outpatient private laboratory, leading to a selection bias. Moreover, individuals referred for CPET evaluation may be more symptomatic than individuals not referred for CPET, which may make the results of our study more applicable to patients with long COVID and thus, a strength rather than limitation of our study. Subjects with critical illness were also underrepresented in our study, and it is possible that a reduction in CRF could be even more pronounced in this subgroup. The interval between assessments varied in the sample with CPET prior to viral infection. However, the calculation of the age-related reductions in predicted peak VO_2_demonstrated that aging could explain approximately 20% of the observed reduction in the variable. Thus, we assume that our findings were mainly related to COVID-19 and not to the time interval between assessments. Finally, although this is a relatively medium-term study (CPET performed at a median of 11.5 weeks after disease onset), a longer follow-up period is needed to better understand long COVID and the clinical impact of intolerance to exercise, as well as the effects exercise training may have on this patient population.

## Conclusions

Peripheral muscle fatigue was the most common cause of exercise limitation in post-COVID-19 patients, regardless of the illness severity, and reductions were observed mainly in VO_2_and HR at peak exercise and ventilatory thresholds. Ventilatory equivalents, OUES, and peak oxygen pulse were not different among illness severity or before-after COVID-19 comparisons. Our data strengthen the importance of using CPET in post-COVID-19 subjects with residual symptoms to help discover the most compromised systems to tailor rehabilitation efforts.
